# Common Origin of the Cerebellar Dual Somatotopic Areas Revealed by Tracking Embryonic Purkinje Cell Clusters with Birthdate Tagging

**DOI:** 10.1523/ENEURO.0251-20.2020

**Published:** 2020-11-12

**Authors:** Khoa Tran-Anh, Jingyun Zhang, Viet Tuan Nguyen-Minh, Hirofumi Fujita, Tatsumi Hirata, Izumi Sugihara

**Affiliations:** 1 Department of Systems Neurophysiology, Graduate School of Medical and Dental Sciences, Tokyo Medical and Dental University, Bunkyo-ku, Tokyo 113-8519, Japan; 2 Center for Brain Integration Research, Tokyo Medical and Dental University, Bunkyo-ku, Tokyo 113-8519, Japan; 3 Department of Otolaryngology-Head and Neck Surgery, Johns Hopkins University, School of Medicine, Baltimore, Maryland 21205, U.S.A.; 4 Brain Function Lab, National Institute of Genetics, Yata, Mishima-shi, Shizuoka-ken 411-8540, Japan

**Keywords:** cerebellum, compartmentalization, Purkinje cell, Purkinje cell cluster, somatosensory, somatotopic representation

## Abstract

One of the notable characteristics of the functional localization in the cerebellar cortex is the dual representation of the body (somatotopy) on its anterior-posterior axis. This somatotopy is conspicuous in the C1/C3 module, which is demarcated as the multiple zebrin-negative and weekly-positive stripes in dual paravermal areas in anterior and posterior lobules within the cerebellar compartments. In this report, we describe the early formation process of the cerebellar compartmentalization, particularly in the C1/C3 module. As developing PCs guide formation of the module-specific proper neuronal circuits in the cerebellum, we hypothesized that the rearrangement of embryonic Purkinje cell (PC) clusters shapes the adult cerebellar compartmentalization. By identifying PC clusters with immunostaining of marker molecules and genetical birthdate-tagging with *Neurog2*-CreER (G2A) mice, we clarified the three-dimensional spatial organization of the PC clusters and tracked the lineage relationships among the PC clusters from embryonic day 14.5 (E14.5) till E17.5. The number of recognized clusters increased from 9 at E14.5 to 37 at E17.5. Among E14.5 PC clusters, the c-l (central-lateral) cluster which lacked E10.5-born PCs divided into six c-l lineage clusters. They separately migrated underneath other clusters and positioned far apart mediolaterally as well as rostrocaudally by E17.5. They were eventually transformed mainly into multiple separate zebrin-negative and weakly-positive stripes, which together configured the adult C1/C3 module, in the anterior and posterior paravermal lobules. The results indicate that the spatial rearrangement of embryonic PC clusters is involved in forming the dual somatotopic areas in the adult mouse paravermal cerebellar cortex.

## Visual Abstract

**Figure:**
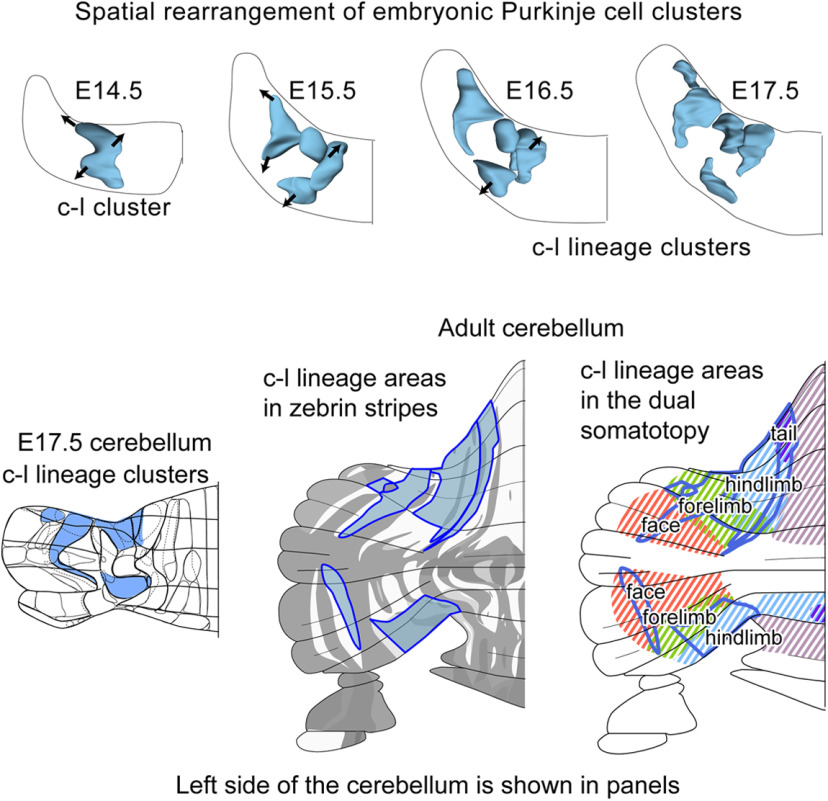

## Significance statement

Genetically programmed morphogenetic processes in the embryonic brain can form a highly organized anatomical complex in the postnatal brain. The adult cerebellum has a complex functional localization; one of the challenging aspects of which is the dual representation of somatosensorimotor function in both the anterior and posterior paravermal areas. To elucidate morphogenetic processes of the intricate organization of the cerebellar cortex, we tracked lineages of early cerebellar PC clusters by birthdate-tagging methods. Starting with nine clusters at embryonic day 14.5, we clarified the differentiation of lineage of all clusters in later stages. Our results indicate that the spatial differentiation of embryonic PC clusters is involved in forming the basic cerebellar organization of the mouse brain.

## Introduction

**Figure 1.**
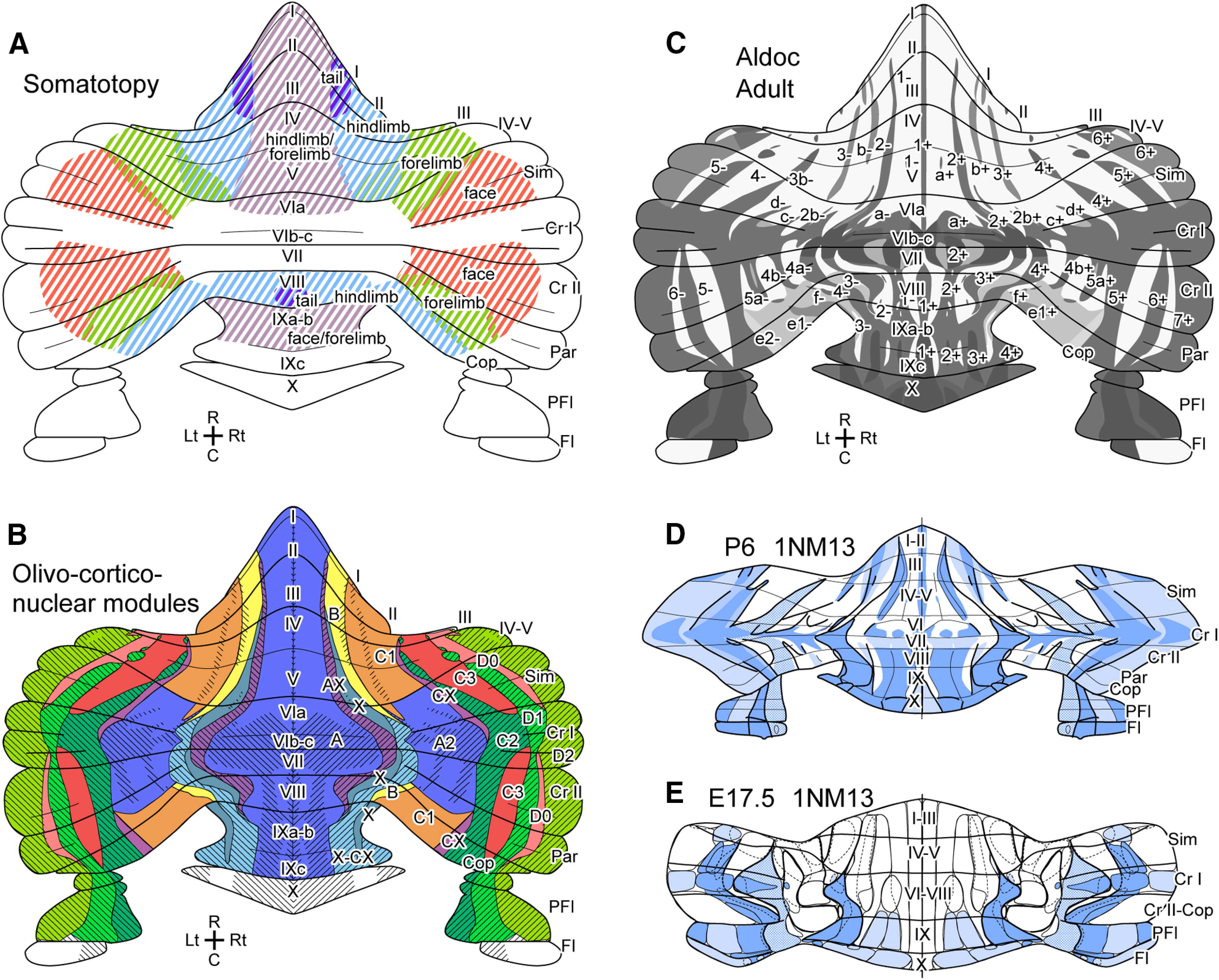
Introductory schematic drawings of the somatotopy and compartments mapped in the unfolded scheme of the mouse cerebellar cortex. ***A***, Somatotopy mainly based on mapping of mossy fiber terminal response (Welker, 1987) and labeled spinocerebellar and cuneocerebellar projections in rodents (Quy et al., 2011; Luo et al., 2018, 2020). ***B***, Olivocorticonuclear modules (colored areas) defined by the topographic axonal projections between subareas of the inferior olive, cerebellar nuclei and cerebellar cortex (A, AX, A2, B, CX, X, X-CX, C1, C2, C3, D0, D1 and D2 modules; Voogd and Glickstein, 1998; Sugihara and Shinoda, 2004; Ruigrok et al., 2015). Shadowed areas belong to aldolase C-positive stripes. ***C***, Stripes defined by aldolase C expression pattern in Purkinje cells (Fujita et al., 2014; Sarpong et al., 2018). ***D*** and ***E***, Mapping of clusters of PCs at E14.5 and immature stripes of PCs at P6 based on Fujita et al. (2012). Blue areas indicate particular clusters or immature stripes that express lacZ in 1NM13 transgenic mice, which often overlap with zebrin stripes. Abbreviations, c-l, c-m, d, dl, l, m, ml, rdl, vl, names of E14.5 clusters; C, caudal; D, dorsal; L, lateral; M, medial; R, rostral. V, ventral.

Representation of the somatotopy is deeply involved in the motor control function in the cerebellum (Manni and Petrosini, 2004). Electrophysiological and neuroimaging studies have shown dual somatotopic areas in the anterior and posterior cerebellar lobules which is one of the noticeable features of the functional localization of the cerebellar cortex of humans and other mammals (Fig. 1*A*; Thickbroom et al., 2003; Manni and Petrosini, 2004).

Morphologically, the cerebellar cortex is organized by multiple longitudinal striped subdivisions. Two types of mutually linked subdivisions, 1) modules and 2) molecular compartments, have been identified. The modules have been defined by the topographic connections of PC axons and climbing fiber axons (Fig. 1*B*; Voogd and Glickstein, 1998; Apps and Hawkes, 2009; Cerminara et al., 2013; Fujita and Sugihara, 2013; Ruigrok et al., 2015), whereas the molecular compartments have been defined by the arrangement of Purkinje cells (PCs) that show heterogeneous expression of marker molecules such as zebrin II or aldolase C (Fig. 1*C*; Brochu et al., 1990; Voogd and Glickstein, 1998; Sugihara and Shinoda, 2004; Sillitoe and Joyner, 2007; Fujita et al., 2014). The somatotopic representation is most clearly seen in the paravermal area in anterior and posterior lobules, in which zebrin-negative and -faintly-positive stripes (identical to the C1/C3 module; Fig. 1*B, C*) occupy substantial proportions of the cerebellar cortex. Both the anterior and posterior parts of this area are topographically innervated by the climbing fiber axons originating from the dorsal accessory olive and project to the anterior interposed nucleus (Ekerot et al., 1997; Cerminara et al., 2013; Ruigrok et al., 2015; Low et al., 2018) to be involved in the control of fine body movements such as grasping and limb cutaneous reflexes in the cat (Horn et al., 2010) and rat (Pijpers et al., 2008). Because the C1/C3 module represents the main part of the cerebellar somatotopic area as mentioned above, the anteroposterior separation of the C1/C3 module (Fig. 1*B*) may be the anatomical correlate for the anteroposterior dual representation of somatotopy observed in animal and human cerebellums (Snider, 1950; Stoodley et al., 2012; Guell et al., 2018).

PCs are born in the period between embryonic day 10.5 (E10.5) and E12.5 in the ventricular zone (Hashimoto and Mikoshiba, 2003) and form the main body of the immature cerebellum by E14.5 in mice (Goffinet, 1983). At E14.5, some eight heterogeneous subsets of PCs are arranged in clustered compartments as observed by molecular marker labeling (Vibulyaseck et al., 2017) or genetic profiling (Wizeman et al., 2019). At E17.5, the number of heterogeneous populations of PCs increases to about 50, which are arranged into clusters separated by PC-free gaps (Fig. 1*E*; Korneliussen, 1968; Altman and Bayer, 1985; Smeyne et al., 1991; Oberdick et al., 1993; Millen et al., 1995; Larouche et al., 2006; Wilson et al., 2011; Fujita et al., 2012; Carter et al., 2018; Wizeman et al., 2019). Each of the E17.5 clusters develops directly into an individual adult PC stripe in the postnatal period (Fig. 1*D*; Sillitoe et al., 2009; Namba et al., 2011; Fujita et al., 2012). Therefore, we hypothesized that the rearrangement of embryonic PC clusters is essential in shaping the compartmental organization of the adult cerebellar cortex which includes its modular organization and the dual somatotopic areas. However, accurate spatial tracking of lineages of all E14.5 clusters would be required to test this hypothesis.

Each striped compartment in the adult cerebellar cortex contains PCs generated at particular timing (Hashimoto and Mikoshiba, 2003; Namba et al., 2011; Zhang et al., 2020). Therefore, birthdate-specific labeling of PCs can be a useful technique to track the cerebellar compartmentalization. The CreER-LoxP system that targets the *ascl1* gene, which is transiently expressed at the time of neuronal differentiation, can label neurons that are born at the time of tamoxifen injection (Sudarov et al., 2011). We used a similar birthdate-tagging system (G2A mouse line, Hirata et al., 2019; Zhang et al., 2020) that targets the *Neurog2* gene, which is expressed in neurons including PCs (Zordan et al., 2008), when the neuronal progenitors start differentiating (Florio et al., 2012).

By combining birthdate-specific labeling and molecular marker labeling of PCs (Minaki et al., 2008; Fujita et al., 2012), we tracked the migration and division of all embryonic PC clusters from E14.5 to E17.5 to clarify the spatial development of the cerebellar compartmentalization. We then focused on the lineage of a particular E14.5 cluster, the fate of which was crucial to test the above hypothesis.

## Materials and Methods

### Ethics statements

Experimental protocols were approved by the Animal Care and Use Committee (A2019-187A, A2018-148A, A2017-060C4) and Gene Recombination Experiment Safety Committee (G2019-020A, 2017-040A, 2012-064C4) of Tokyo Medical and Dental University.

### Animals

Mice were bred and reared in a 12-12-hour light-dark cycled condition in the animal facility of the university with freely available food and water. Wild-type embryo samples were obtained by mating B6C3F1 males and females. The C57BL/6N-Tg(Neurogenin2-CreER) mouse strain (G2A, deposited at RIKEN BDRAccession No. CDB0512T−1, http://www2.clst.riken.jp/arg/TG%20mutant%20mice%20list.html, Hirata et al., 2019) has the transgene, in which CreER gene has been inserted into the downstream side of the enhancer region of neurogenin2 gene (*Neurog2*), presumptively on the Y chromosome (Hirata et al., 2019). Since the CreER is expressed in differentiating PCs after the last mitosis under the *Neurog2* enhancer (Florio et al., 2012), administration of tamoxifen, a ligand of the estrogen receptor, produces Cre activity in cells in which *Neurog2* is expressed in G2A mice. In B6.Cg-Gt(ROSA)26Sor^tm9(CAG-tdTomato)Hze^/J:C57BL/6N mice (Ai9, The Jackson Laboratory, https://www.jax.org/strain/007), Cre activity produces a persistent tdTomato expression in cells. Male heterozygous G2A mice were crossed with female homozygous Ai9 mice to produce G2A::Ai9 embryos. The day when the vaginal plug was detected was designated as E0.5. Tamoxifen (T5648-1G, Sigma, St. Louis, MO, U.S.A.) was dissolved in corn oil (9 mmole/l, 032-17016, Wako, Wako Pure Chemical Industries, Ltd., Osaka, Japan) and injected intraperitoneally (2.25 μmole/mouse) one time to the pregnant female at noon, 10, 11 or 12 days after the plug detection (at E10.5, E11.5, E12.5). In AldocV mice (MGI:5620954, Fujita et al., 2014), aldolase C (zebrin II) stripes are labeled by a mutated green fluorescent protein, Venus. We produced double-homozygous Ai9::AldocV mice. An Ai9::AldocV double-homozygous female was crossed with a G2A heterozygous male (Zhang et al., 2020). Tamoxifen was injected into the pregnant female as above. E19.5 embryos were obtained by Caesarean section from pregnant Ai9::AldocV double homozygous females which were sacrificed beforehand by cervical dislocation. Male pups (G2A::Ai9::AldocV heterozygous hybrid mice) were reared by a stepmother and perfused at postnatal day 42.

### Histological procedures

A Caesarean section was performed on pregnant Ai9 females anesthetized with an intramuscular injection of pentobarbital sodium (0.1 mg/g body weight) and xylazine (0.005 mg/g body weight) to obtain E14.5-E17.5 embryos at noon. Embryos were perfused transcardially with phosphate-buffered saline (PBS, pH 7.4) with heparin sulfate (0.1%), and then with 4% paraformaldehyde. The anesthetized female was sacrificed by cervical dislocation after removing embryos. The embryo brains were dissected in chilled 4% paraformaldehyde and kept in 4% paraformaldehyde for post-fixation and then soaked in sucrose solution (30% with 0.05M phosphate buffer, pH 7.4) for two days. Among embryo samples obtained from pregnant Ai9 female, ones that showed patterned red fluorescence reporter expression in the brain were regarded as G2A::Ai9 heterozygous hybrid. Brain samples were stored in the deep freezer until sectioning.

G2A::Ai9::AldocV heterozygous hybrid mice were anesthetized with an intramuscular injection of an overdose of pentobarbital sodium (0.18 mg/g body weight) and xylazine (0.009 mg/g body weight) at postnatal day 42. They were perfused transcardially with PBS with 0.1% heparin sulfate, and then with 4% paraformaldehyde. The skull was kept in 4% paraformaldehyde for post-fixation overnight. The brain was dissected and soaked in sucrose solution for two days. Brain samples were stored in the deep freezer until sectioning.

Embryo G2A::Ai9 brains were coated with gelatin solution (10% gelatin, 10% sucrose in 10mM phosphate buffer, pH 7.4, 32°C). The gelatin block was hardened by chilling and then soaked overnight in fixative with a high sucrose content (4% paraformaldehyde, 30% sucrose in 0.05 M phosphate buffer, pH 7.4). Complete sets of serial sections were cut coronally, horizontally and sagittally using freezing microtome at a thickness of 40 μm. The ventral surface of the medulla was regarded as the horizontal plane. After washing in PBS and PBS with 0.12% Triton X-100 (PBST), each complete set of sections was processed for immunostaining. Floating sections were incubated on a shaker with a mixture of two or three primary antibodies produced in different host animal species in PBST plus 2% normal donkey serum for 48 hours at 4°C. Goat anti-EphA4 (R&D Systems), goat anti-FoxP2 (Everest Biotech), rabbit anti-Corl2 (provided by Dr. Ono at KAN Research Institute) and rat anti-OL-protocadherin (Millipore) are the primary antibodies used in the majority of experiments. Rabbit anti-FoxP2 antibody (Abgent) was used in combination with the goat primary antibody. The specificity of the above antibodies has been described (Vibulyaseck et al., 2017). In some experiments, mouse anti-Calbindin-D28k (Sigma-Aldrich) and rabbit anti-Calbindin-D28k (AnaSpec) antibodies were also used (Table 1). The sections were then incubated with a mixture of appropriate two or three secondary antibodies that were conjugated with fluorescent tags (Table 1). Some sections were counterstained with 4’, 6-diamidino-2-phenylindole dihydrochloride (DAPI; 1:3,000; D212, Dojindo, Mashiki, Kumamoto, Japan). Finally, these sections were mounted on glass slides, dried, coverslipped with water-soluble mounting medium (CC mount, Sigma C9368-30ML).

**Table 1:** Antibodies used in the study

	**Antigen**	**Manufacturer, species, mono- or** **polyclonal, catalog or lot No., RRID**	**Concentration**
Primary Antibodies	Corl2	Dr. Yuichi Ono (KAN Research Institute), rabbit polyclonal, affinity-purified	1:350
	EphA4	R&D Systems, goat polyclonal, Cat# AF641, Lot #BVX0308091	1:1000
	FoxP2	Everest Biotech (Oxfordshire, UK), goat polyclonal, Cat# EB05226, Lot # 160409, RRID: AB_2107112	1:5000
	FoxP2	Abgent, rabbit polyclonal, Cat# AP5753b, Lot #SA100916AA, RRID: AB_10818782	1:1000
	Pcdh10	Millipore, rat monoclonal, clone 5G10, Cat# MABT20, Lot # NRG1759424, RRID:AB_10807416	1:1600
	Calbindin D28	Sigma, mouse monoclonal, Cat# 175651C8666	1:500
Secondary Antibodies	Anti-Goat IgG, Alexa Fluor 488	Jackson ImmunoResearch, donkey, Cat# 705-545-147	1:400
	Anti-Goat IgG, Alexa Fluor 680	Jackson ImmunoResearch, donkey, Cat# 705-625-147	1:400
	Anti-Rabbit IgG, Alexa Fluor 405	abcam, donkey, Cat# 175651	1:500
	Anti-Rabbit IgG, Alexa Fluor 488	Jackson ImmunoResearch, donkey, Cat# 711-545-152	1:400
	Anti-Rabbit IgG, Alexa Fluor 647	Jackson ImmunoResearch, donkey, Cat# 711-605-152	1:400
	Anti-Rabbit IgG, Alexa Fluor 594	Jackson ImmunoResearch, donkey, Cat# 711-585-152	1:400
	Anti-Rabbit IgG, Teas Red	Jackson ImmunoResearch, donkey, Cat# 711-075-152	1:200
	Anti-Rat IgG, DyLight 594	Jackson ImmunoResearch, donkey, Cat# 712-515-153	1:200
	Anti-Rat IgG, Alexa Fluor 647	Jackson ImmunoResearch, donkey, Cat# 705-605-150	1:400
	Anti-Rat IgG, Alexa Fluor 680	abcam, donkey, Cat# 175777	1:500
	Anti-Mouse IgG, Alexa Fluor 647	Jackson ImmunoResearch, donkey, Cat# 715-605-150	1:400

Postnatal G2A::Ai9::AldocV brains were embedded in gelatin, and cut coronally into serial sections at a thickness of 50 μm. The complete sets of sections were mounted on glass slides, dried, coverslipped with water-soluble mounting medium (CC mount).

### Acquisition of digital images

Fluorescent images were digitized using a cooled color CCD camera (AxioCam1Cm1, Zeiss, Oberkochen, Germany) attached to a fluorescent microscope (AxioImager.Z2, Zeiss) in 12-bit gray-scale with appropriate filter sets. To digitize a section of the cerebellum, 2.5X objective and tiling function of the software to control digitizing (Zen 2 Pro, Zeiss) was used. Images of all serial sections of a brain were obtained with the same exposure and gain parameters. Images were adjusted in contrast and brightness and assembled using a software (ZEN 2 Pro, Zeiss and Photoshop 7, Adobe, San Jose, CA, USA). High magnification confocal images were taken with a 63X objective lens and appropriate filters and laser light sources attached to the confocal microscope (TCS SP8, Leica, Wetzlar, Germany). Images were adjusted in contrast and brightness and assembled using software (Las X, Leica). A combination of pseudo-colors was applied to fluorescent and confocal images in figures. Photographs were assembled using Photoshop and Illustrator software (Adobe). Digital enhancements were applied to entire images and no manipulations were applied other than contrast or brightness.

### Three-dimensional reconstruction of Purkinje cell clusters

Three-dimensional (3D) models of PC clusters were reconstructed through two steps: 1) two dimensional (2D) drawings of contours of identified PC clusters, and 2) 3D surface modeling with these 2D drawings. Digital images of serial sections were placed in individual layers of 2D graphic software (Adobe Illustrator 10). Their positions and orientations were then adjusted by superimposing them on each other while referring to landmark structures such as the midline, the cerebellar surface and major labeled areas. The cerebellar surface and contour of groups of Purkinje cell subsets and marker-labeled areas (i.e. PC cluster) were drawn using curve tools of Illustrator in all sections. Distribution patterns of PCs and PC-free gaps, and expression patterns of PC markers were systematically observed in this procedure. Cerebellar nuclear areas identified by the lack of Corl2 signal were excluded from the reconstruction. After the identification of PC clusters, all drawings in sections of coronal, horizontal and sagittal planes were imported into 3D graphics software (Rhinoceros 4, Robert McNeel & Associates, Seattle, WA, USA), with the z-axis position adjusted for each section. The 3D drawings of cerebellar structures obtained from brain samples with different cutting orientations were matched and compared with one another to identify structures. The 3D surface reconstructions were made from a set of coronal section drawings by using the ‘loft’ command in Rhinoceros (Fujita et al., 2012). Cerebellar fissures were reconstructed in the 3D space from aligned drawings from sagittal sections.

### Definition of the relative position of a coronal plane within the cerebellum

The relative position of a section in the whole extent of the cerebellum was defined using percentages as described previously (Vibulyaseck et al., 2017). In short, the position of the most caudal section of the coronal sections in which the cerebellum first appeared was defined as 0%, whereas the position of the most rostral section in which the cerebellum remained was defined as 100%. The position of other sections was obtained by linear interpolation. In the case of horizontal sections, the most dorsal section was defined as 0%, while the most ventral section in which the cerebellum remained was defined as 100%.

## Results

### Spatial organization of PC clusters in the embryonic cerebellums at E14.5, E15.5, E16.5, and E17.5

Although embryonic PC clusters were suggested to be the direct origin of adult cerebellar compartments (Fujita et al., 2012), the development of embryonic PC clusters has not been fully clarified before E17.5 except for the Pcdh10-positive areas which have been tracked in our previous study (Vibulyaseck et al., 2017). In the first set of experiments in the present study, we identified PC clusters in the entire cerebellum at a 1-day interval from E14.5 to E17.5. The distribution of PCs was analyzed by examining the expression of particular molecular markers and PC-free gaps in serial coronal, horizontal and sagittal sections in 22 wild-type B6C3F1 mouse embryos (E14.5, n=7; E15.5, n=5, E16.5, n=5, E17.5, n=5). Expression of FoxP2 (relatively specific PC marker, Fujita et al., 2012; Vibulyaseck et al., 2017), Corl2 (also known as Skor2, specific PC marker, Minaki et al., 2008; Vibulyaseck et al., 2017), Pcdh10 (PC cluster marker, Fujita et al., 2012; Vibulyaseck et al., 2017) and EphA4 (PC cluster marker expressed in PCs and afferent axons, Fujita et al., 2012; Vibulyaseck et al., 2017) were immunohistochemically revealed.

**Figure 2.**
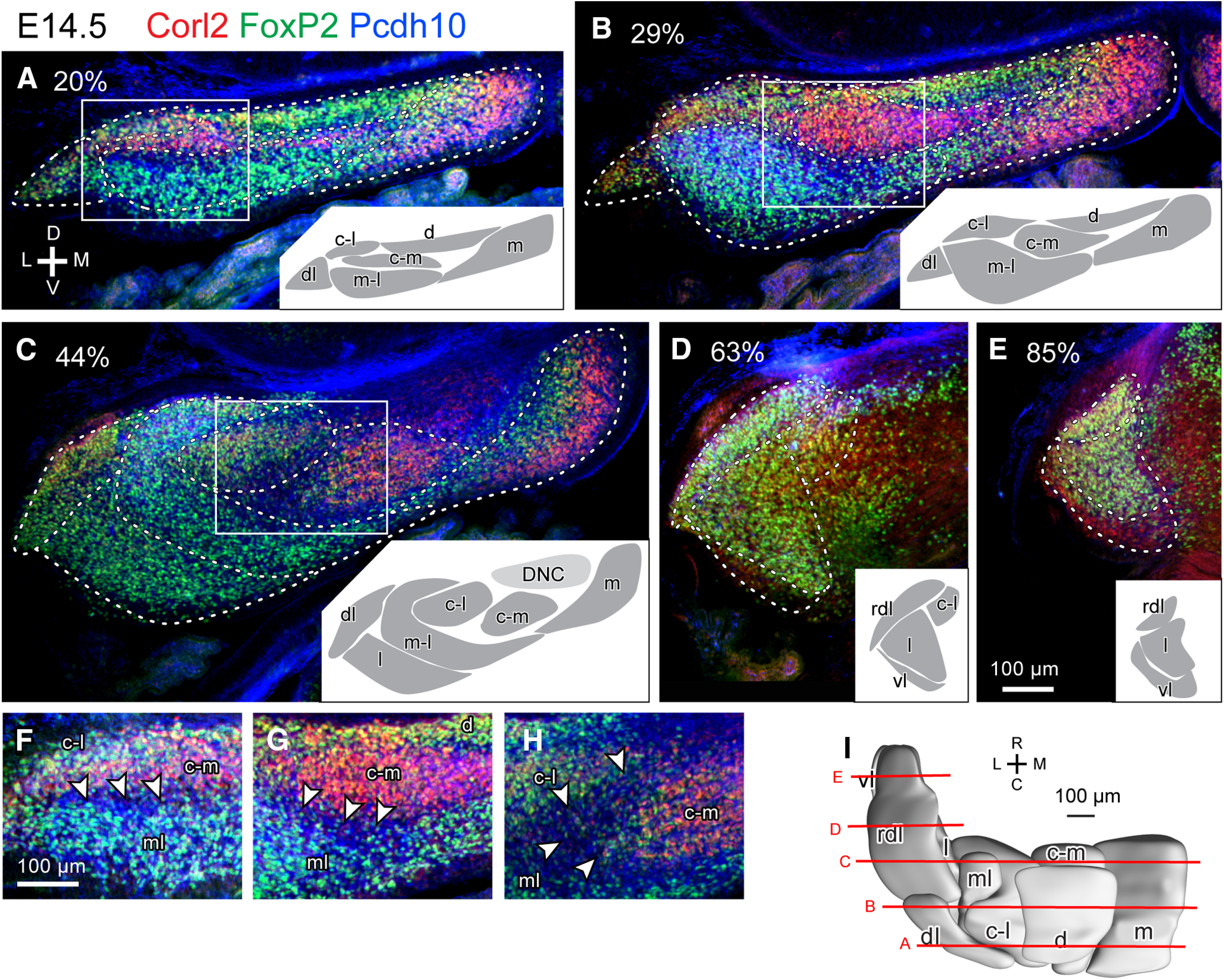
Purkinje cell clusters in coronal sections of the left E14.5 mouse cerebellum identified by marker expression profiles. ***A–E***, Sections at different caudorostral levels indicated by percentile. Fluorescent signals of immunostaining for Corl 2 (red), Pcdh10 (blue) and FoxP2 (green) are merged. White dashed lines indicate the boundary of recognized clusters. Inset in each panel shows drawings of recognized clusters at half magnification. Squares indicate areas that are magnified. ***F–H***, Magnified images showing PC-free gaps (arrowheads) between some clusters. ***I***, Dorsal view of the 3D reconstruction of clusters. Red lines indicate the rostrocaudal level of the section in each panel. Scale bar in E (100 μm) applies to ***A–E***. Scale bar in F (100 μm) applies to ***F–H***. Abbreviations, c-l, c-m, d, dl, l, m, ml, rdl, vl, names of E14.5 clusters; C, caudal; D, dorsal; DCN, deep cerebellar nucleus; L, lateral; M, medial; R, rostral.V, ventral.

**Figure 3.**
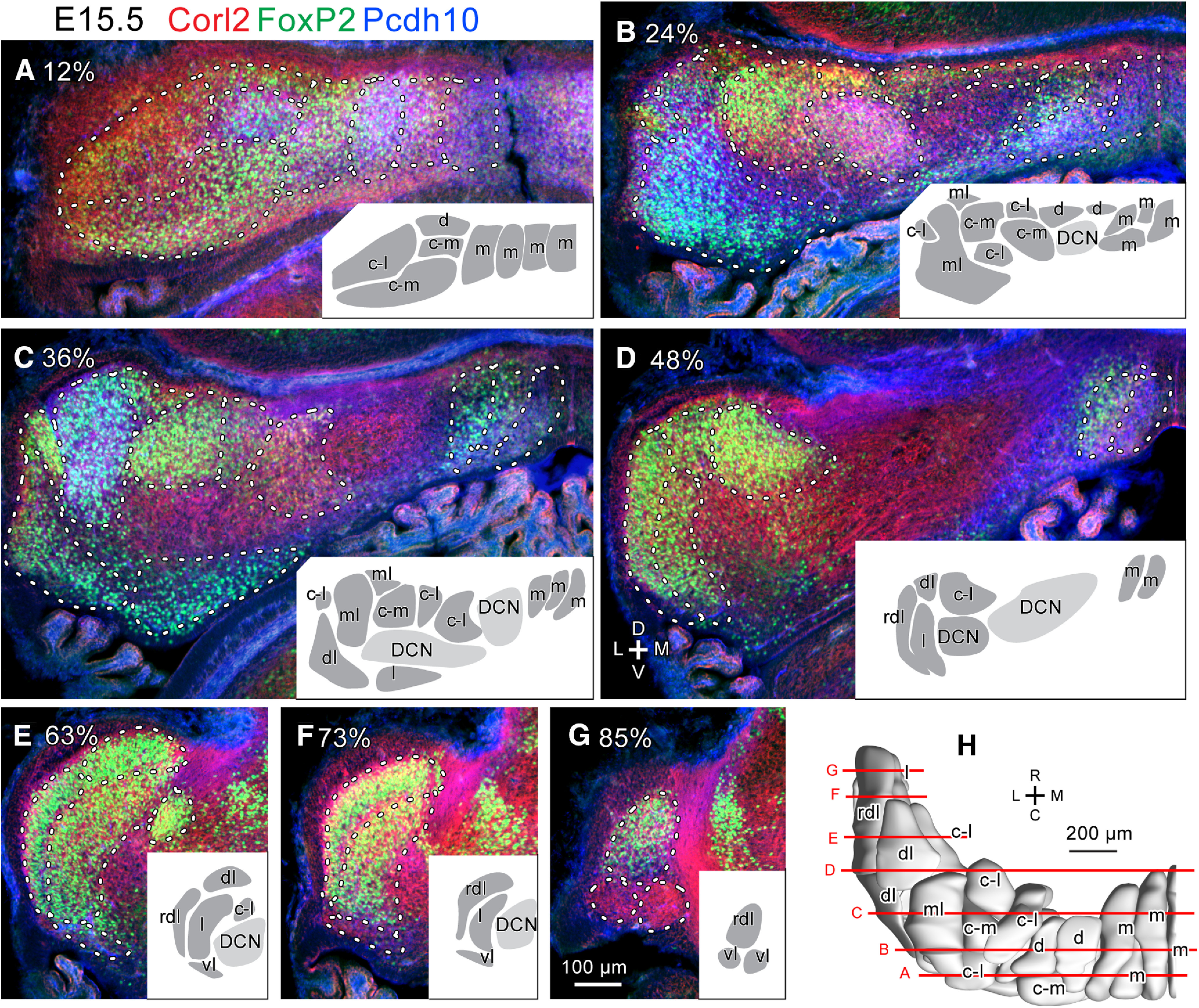
Purkinje cell clusters in coronal sections of the left E15.5 mouse cerebellum identified by marker expression profiles. ***A–G***, Sections at different caudorostral levels indicated by percentile. Fluorescent signals of immunostaining for Corl 2 (red), Pcdh10 (blue) and FoxP2 (green) are merged. White dashed lines indicate the boundary of recognized clusters. Inset in each panel shows drawings of recognized clusters at half magnification. Names of each cluster were given later based on the lineage analysis (c.f. Fig. 7). ***H***, Dorsal view of the 3D reconstruction of clusters. Red lines indicate the rostrocaudal level of the section in each panel. Scale bar in G (100 μm) applies to panels ***A–G***. Abbreviations, c-l, c-m, d, dl, l, m, ml, rdl, vl, names of the lineage of clusters; C, caudal; D, dorsal; DCN, deep cerebellar nucleus; L, lateral; M, medial; R, rostral.V, ventral.

**Figure 4.**
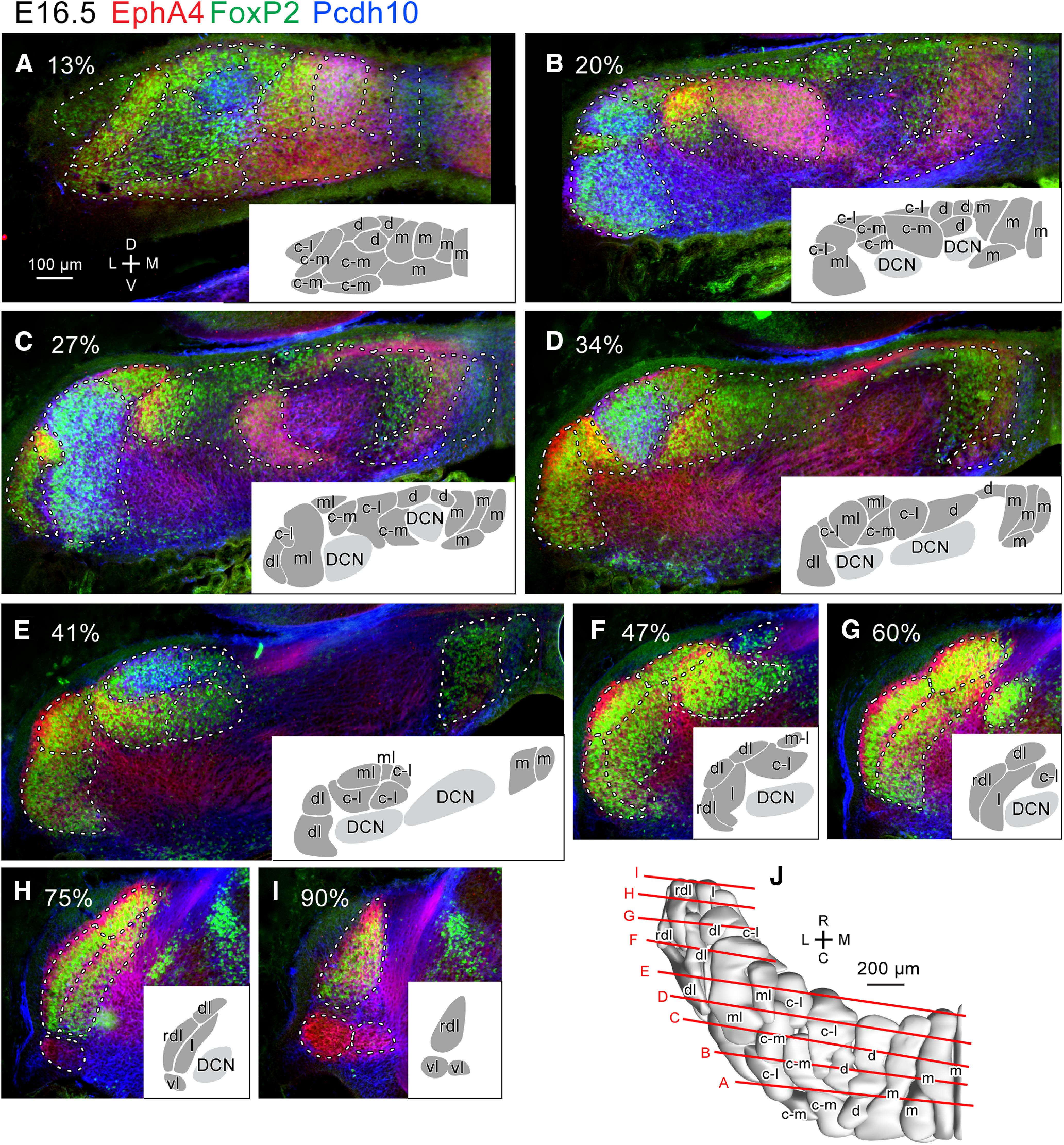
Purkinje cell clusters in coronal sections of the left E16.5 mouse cerebellum identified by marker expression profiles. ***A–I***, Sections at different caudorostral levels indicated by percentile. Fluorescent signals of immunostaining for EphA4 (red), Pcdh10 (blue) and FoxP2 (green) are merged. White dashed lines indicate the boundary of recognized clusters. Inset in each panel shows drawings of recognized clusters at half magnification. Names of each cluster were given later based on the lineage analysis (c.f. Fig. 7). ***J***, Dorsal view of the 3D reconstruction of clusters. Red lines indicate the rostrocaudal level of the section in each panel. Scale bar in A (100 μm) applies to panels ***A–I***. Abbreviations, c-l, c-m, d, dl, l, m, ml, rdl, vl, names of the lineage of clusters; C, caudal; D, dorsal; DCN, deep cerebellar nucleus; L, lateral; M, medial; R, rostral.V, ventral.

**Figure 5.**
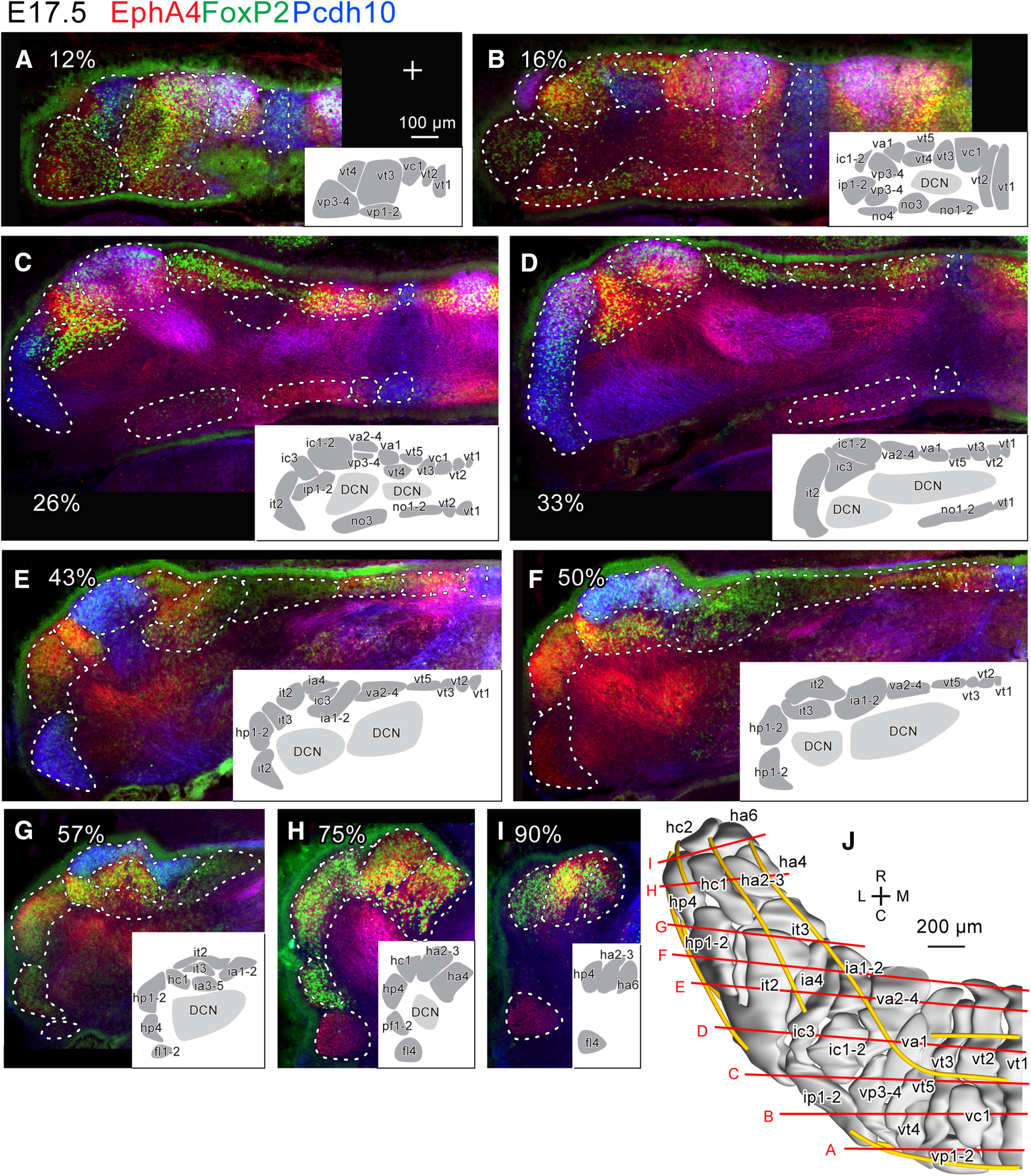
Purkinje cell clusters in coronal sections of the left E17.5 mouse cerebellum identified by marker expression profiles. ***A–I***, Sections at different caudorostral levels indicated by percentile. Fluorescent signals of immunostaining for EphA4 (red), Pcdh10 (blue) and FoxP2 (green) are merged. White dashed lines indicate the boundary of recognized clusters. Inset in each panel shows drawings of recognized clusters at half magnification. Names of each cluster were basically adopted from Fujita et al. (2012), but see Results. ***J***, Dorsal view of the 3D reconstruction of clusters. Yellow lines indicate immature fissures. Red lines indicate the rostrocaudal level of the section in each panel. Scale bar in ***A*** (100 μm) applies to panels ***A–I***. Abbreviations, fl1-2, fl4, ha2-3, ha4, ha6, hc1, hc2, hp1-2, hp4, ia1-2, ia3-5, ia4, ic1-2, ic3, ip1-2, it2, it3, no1-2, no3, no4, pf1-2, va1, va2-4, vc1, vp1-2, vp3-4, vt1, vt2, vt3, vt4, vt5, names of E17.5 clusters; C, caudal; D, dorsal; DCN, deep cerebellar nucleus; L, lateral; M, medial; R, rostral.V, ventral.

We compared the labeling pattern between left and right sides and among different samples cut in coronal, horizontal and sagittal sections at each embryonic date. Interindividual variations, such as those in the marker expression level, shape, size, and positional relationships of clusters were small. Thus, we comprehensively identified PC clusters at E14.5 (Fig. 2), E15.5 (Fig. 3), E16.5 (Fig. 4), and E17.5 (Fig. 5), and confirmed previously identified PC clusters at E14.5 (Vibulyaseck et al., 2017) and E17.5 (Fujita et al., 2012). We then reconstructed identified PC clusters of E14.5, E15.5 and E16.5 cerebellums in the three-dimensional space (Figs. 2*I*, 3*H*, 4*J*) primarily based on images of immunostaining on one side of serial coronal sections. For those of E17.5, the previously published reconstruction (Fujita et al., 2012) was incorporated with some revisions (see below; Fig. 5*J*).

At E14.5, nine PC clusters—termed medial, dorsal, central-medial, central-lateral, mid-lateral, lateral, dorsolateral, rostrodorsolateral, and ventrolateral (m, d, c-m, c-l, ml, l, dl, rdl, and vl; based on Vibulyaseck et al., 2017)—were arranged in column-shaped elongations in the rostrocaudal direction at various mediolateral and dorsoventral levels (Fig. 2*I*). A previously designated Pcdh10-positive cluster, termed “c” (Vibulyaseck et al., 2017), was revised here: it was divided into the central-medial (c-m) and central-lateral (c-l) clusters because the expression of Corl2 was stronger in the c-m than in the c-l (Fig. 2*A–C*). The distribution of these nine clusters was remarkably consistent with that of the unbiasedly classified groups of PCs via single-cell RNAseq (Wizeman et al., 2019; see Discussion). These clusters, thus, may reflect fundamental molecular distinctions in PCs at E14.5.

The number of PC clusters increased from 9 at E14.5 (Fig. 2), to 18 at E15.5 (Fig. 3), and to 28 at E16.5 (Fig. 4). The number increased presumably because 1) new PC-free gaps appeared inside a cluster, and/or 2) a part of a cluster changed in molecular expression from the other part of the same cluster. Furthermore, some divided clusters seemed to migrate separately. At E17.5, PC clusters were narrow in the mediolateral direction but often extended rostrocaudally (longitudinally) across immature lobules, somewhat resembling adult striped compartments, although they were not yet arranged in a single layer but in multiple layers, shallow or deep from the cerebellar surface (Fig. 5; Fujita et al., 2012).

Although E17.5 cerebellum contains 54 clusters identified with detailed analyses (Fujita et al., 2012), this study focused on the more qualitative distinction of clusters to facilitate analyses and simplify description. Namely, neighboring clusters that had only slightly different molecular expression profiles and/or not clearly separated from one another by intercalating PC-free gaps were combined. For example, our cluster vp1-2 includes clusters vp1 and vp2 of Fujita et al. (2012). We combined 11 sets of two or three neighboring E17.5 clusters into single clusters, resulting in a total of 37 clusters in place of 54. We adopted the nomenclature (Table 3) from Fujita et al. (2012) to designate E17.5 clusters in the present study.

### Birthdate-specific labeling of PCs in the E14.5 cerebellum

**Figure 6.**
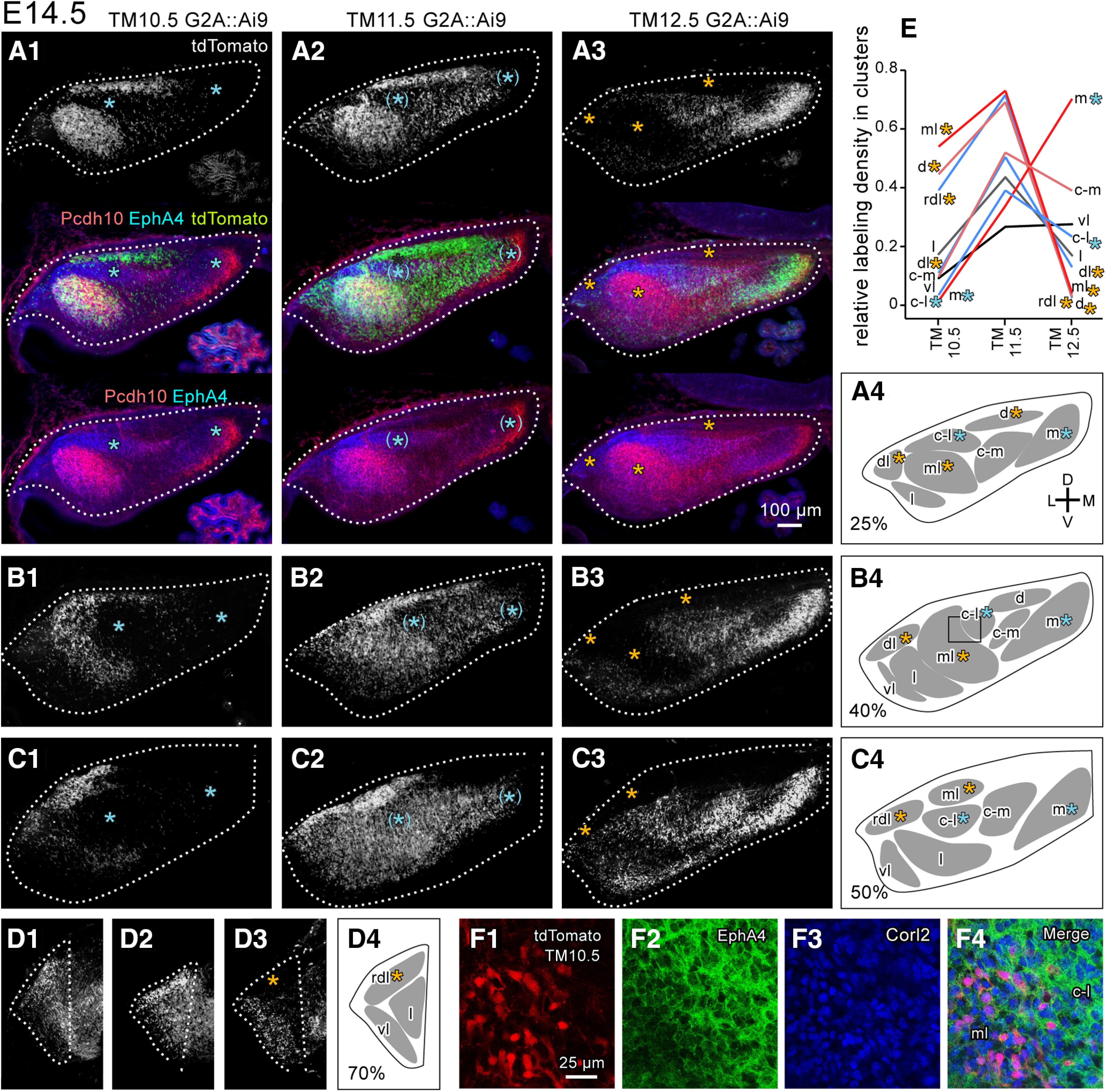
Spatial distribution of E10.5-, E11.5-, and E12.5-born PCs in the E14.5 cerebellum. ***A–D***, Images of coronal sections of the left cerebellum of G2A::Ai9 embryos which had tamoxifen injection at E10.5 (subpanel 1), E11.5 (subpanel 2) and E12.5 (subpanel 3) at different rostrocaudal levels. The top, center and bottom panels show images of the tdTomato fluorescence signal (top), three signals merged, and signals of tdTomato fluorescence (green) and immunostaining signals of Pcd10 (red) and EphA4 (blue) in (***A***), while only the tdTomato fluorescence signal is shown in ***B–D***. The tdTomato signal indicates neurons (mostly PCs) that expressed *Neurog2*-CreER at the time of tamoxifen injection. White dashed lines indicate the coutour of the cerebellum. Subpanel 4 shows schematic drawings of identified clusters. Blue and Orange asterisks indicate (position of) the E10.5-PC-sparse and E12.5-PC-sparse clusters, respectively. ***E***, Relative labeling density in nine E14.5 clusters. The plotted data were the average of the tdTomato fluorescence signal in the digital file (0-255) measured in 9-18 square areas of 900 μm^2^ located inside the identified cluster in 3-6 sections. The same brightness and contrast adjustment was done in all sections. ***F***, Confocal high-magnification image of immunostaining and tdTomato expression at the junction between the ml and c-l clusters in the AM10.5 G2A::Ai9 embryo, showing expression of tdTomato (E10.5-born neurons) colocalized exactly with Corl2 (PC marker) at the cellular level in the ml cluster. The area of this image is indicated in ***B4*** with a square. Abbreviations, c-l, c-m, d, dl, l, m, ml, rdl, vl, names of E14.5 clusters; C, caudal; D, dorsal; L, lateral; M, medial; R, rostral.V, ventral.

In the second set of experiments, we labeled PCs in the birthdate-specific way to identify the lineage of PC clusters. The G2A mouse line expressed tamoxifen-inducible Cre recombinase activity under the transcription control of proneural gene, neurogenin 2 (*Neurog2*). We crossed female Cre-reporter mice (Ai9) with male heterozygous G2A mice so that tamoxifen injection into the pregnant dam at a specific developmental stage of E10.5, E11.5 and E12.5 (designated as TM10.5, TM11.5 and TM12.5) enabled timed activation of Cre recombinase that initiates reporter (tdTomato) expression in PCs in a birthdate-specific way (Fig. 6*A–D*). G2A::Ai9 hybrid embryo brain samples were collected between E14.5 and E17.5 (n=23 total: E14.5-TM10.5, n=1; E14.5-TM11.5, n=1; E14.5-TM12.5, n=1; E15.5-TM10.5, n=2; E15.5-TM11.5, n=1; E15.5-TM12.5, n=3; E16.5-TM10.5, n=3; E16.5-TM11.5, n=1; E16.5-TM12.5, n=3; E17.5-TM10.5, n=3; E17.5-TM11.5, n=2; E17.5-TM12.5, n=2), cut into serial sections and immunostained for EphA4, Pcdh10, and either FoxP2 or Corl2. Combined labeling of EphA4 and Pcdh10 helped to recognize the clusters that were identified in the analyses described in the preceding section (Figs. 2-5). No clear difference was observed in the PC cluster organization between G2A::Ai9 mice with C57BL/6 background and wild type mice with B6C3F1 background.

Tamoxifen administration labeled neurons with tdTomato in the cerebellum of G2A::Ai9 mice (Fig. 6*A–D*). The labeling pattern was dependent on the timing of tamoxifen administration but consistent among cases that had the same administration timing. We first checked the specificity and efficiency of the labeling in the E14.5 cerebellum. All tdTomato-expressing cells (100%) inside a PC cluster coexpressed Corl2 (Fig. 6*F*), in all mice (n=23), indicating that tdTomato labeling was specific to PCs. Efficiency of the labeling was estimated by counting the number of tdTomato-expressing PCs among all PCs. Because the majority of PCs are born between E10.5 and E12.5, the sum of the ratios of the tdTomato-labeled PCs in the TM10.5, TM11.5, and TM12.5 cerebellums would become close to 100% if the efficiency is high. Indeed, in the 10,000-μm^2^ area of dense PC distribution within the ml cluster of TM10.5, TM11.5, and TM12.5 cerebellums (n=1 each) at E14.5, 41.1% (44 PCs out of 107, 44/107), 52.0% (52/100), and 4.7% (5/106) of PCs were tdTomato-positive, respectively, indicating an estimated efficiency of 97.8%. A similar measurement in the c-l cluster showed 1.1% (1/89), 40.8% (40/98) and 45.2% (42/93) labeled PCs in TM10.5, TM11.5 and TM12.5 cerebellums (n=1 each), respectively, at E14.5, indicating an estimated efficiency of 87.1%. The results indicated that the recombination is highly efficient as well as highly dependent on the timing of tamoxifen administration. Similar efficiency and timing-dependency have been also observed in PCs in the adult cerebellum (Zhang et al., 2020). Thus, the PCs labeled by the tamoxifen injection on a specific timing were designated as “E10.5-born” etc. and the area or cluster in which about 1% of PC were labeled with tamoxifen injection at E10.5 was designated as “E10.5-PC-sparse”.

We then examined the distribution of E10.5-born, E11.5-born and E12.5-born PCs in the PC clusters, which were identified as described in the preceding section, in the E14.5 cerebellum. (Fig. 6*A–D* and Table 2). E10.5-born PCs were observed densely in the d, ml, and rdl clusters, moderately or sparsely in the c-m, l, dl, and vl clusters (Fig. 6*A1, B1, C1, D1*). However, almost none of the E10.5-born PCs were observed in the m and c-l clusters (blue asterisks in Fig. 6*A1, B1, C1*). E11.5-born PCs were observed in all clusters (Fig. 6*A2, B2, C2, D2*). They were more densely distributed in the d, ml, and rdl clusters than in other clusters, and were absent in the medial part of the m cluster. E12.5-born PCs distributed densely in the m cluster and moderately in the c-m, c-l, l, and vl clusters (Fig. 6*A3*, *B3*, *C3*, *D3*), but rarely contributed to either the d, ml, dl or rdl clusters (orange asterisks in Fig. 6*A3, B3, C3, D3*). This observation was further quantified by measuring the fluorescence signal intensity, which was supposed to be approximately linearly related to the density of labeled PCs, in each cluster, in digital images of sections of TM10.5, TM11.5 and TM12.5 cerebellums (Fig. 6*E*). The labeling densities of the c-l and m clusters were near 0, lower than those of other clusters in the TM10.5 cerebellum. but increased to higher levels in the TM11.5 and TM 12.5 cerebellums. On the contrary, the labeling densities of the ml, d, and rdl clusters were higher than those of other clusters in the TM10.5 and TM11.5 cerebellums, but decreased to the level near 0, lower than those of other clusters in the TM12.5 cerebellum (Fig. 6*E*).

**Table 2:** Nine E14.5 PC clusters, their molecular expression profiles, PC birthdates and fates at E17.5

E14.5 PC cluster		m	d	c-m	c-l	ml	dl	rdl	l	vl
Molecular expression profile	Corl2	+ ∼ +++	++	+++	++	+	+++	+++	++	++
	Pcdh10	- ∼ ++	++	+ ∼ +++	+/-	+++	+/-	++	+	+/-
	EphA4	- ∼ ++	-	++	++	+++	++	+++	++	+/-
	FoxP2	+ ∼ +++	+++	+	++	+++	++	+++	++	-
PC birthdate		-E11.5,E12.5	E10.5, E11.5, -	E10.5, E11.5, E12.5	- E11.5, E12.5	E10.5, E11.5 -	E10.5, E11.5 -	E10.5, E11.5 -	E10.5, E11.5, E12.5	E10.5, E11.5, E12.5
Fate at E17.5 (E17.5 cluster)		vt1, vt2, vc1, vp1-2, vt3, no1-2	vt4, vt5, va1	vp3-4, ic1-2, no4, no5, no3, ic3	va2-4, ip1-2, ia1-2, it3, ha4, ia3-5	it2, ia4	hp1-2, hc1, ha1, ha2-3	hp3, hp4, pf	ha5, ha6, hc2	fl1-2, fl3, fl4, fl5

Within each cluster excepting the m cluster, PCs that were born on a particular birthdate appeared to distribute randomly. Boundaries of different labeling density areas matched with the boundaries of the clusters defined by marker expression profiles and PC-free gaps in the TM10.5, TM11.5 and TM12.5 cerebellums (Fig. 6*A1–A3*, center subpanels, asterisks). Each E14.5 cluster was composed of the PCs of two or more particular birthdates (Table 2).

In sum, distribution patterns of birthdate-specific PCs were tightly linked with the PC clusters at E14.5. Consequently, the birthdate-specific labeling of PCs were expected to be a useful tool to track the lineage of the 14.5 PC clusters.

### Tracking birthdate-specific subsets of PCs from E14.5 to E17.5

**Figure 7.**
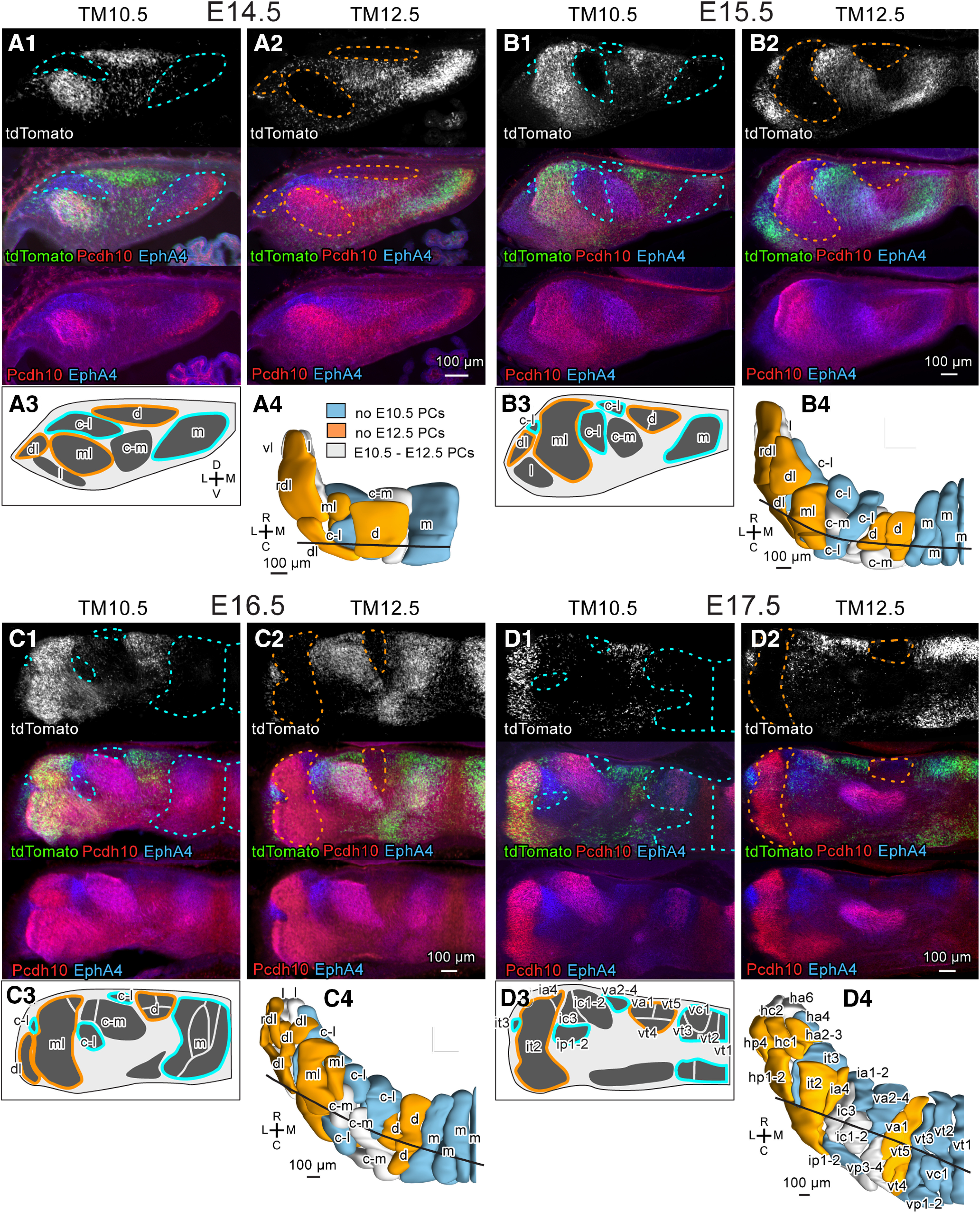
Identification of E10.5-PC-sparse and E12.5-PC-sparse clusters in the E14.5-E17.5 cerebellums. ***A–D***, Images of coronal sections of the left TM10.5 and TM12.5 cerebellums at nearly the same level (subpanel 1 and 2, respectively) and schematic drawing of clusters (subpanel 3) and dorsal view of the 3D scheme (subpanel 4) of the E14.5 (***A***), E15.5 (***B***), E16.5 (***C***), and E17.5 (***D***) cerebellums. In subpanels 1 and 2, the tdTomato fluorescence signal (top), three signals merged, and signals of tdTomato fluorescence (green) and immunostaining signals of Pcd10 (red) and EphA4 (blue) are shown from the top to the bottom. Blue and orange colors in dashed lines (in subpanels in 1 and 2), circumscribing lines (in subpanels 3), and clusters in the 3D schemes (in subpanels 4) indicate E10.5-PC-sparse and E12.5-PC-sparse clusters. Black line in the 3D scheme indicates the position of the coronal section in subpanels 1-3. Abbreviations, c-l, c-m, d, dl, l, m, ml, rdl, vl, names of E14.5 clusters; ha2-3, ha4, ha6, hc1, hc2, hp1-2, hp4, ia1-2, ia4, ic1-2, ic3, ip1-2, it2, it3, va1, va2-4, vc1, vp1-2, vp3-4, vt1, vt2, vt3, vt4, vt5, names of E17.5 clusters; C, caudal; D, dorsal; L, lateral; M, medial; R, rostral.V, ventral.

Since the m and c-l clusters were E10.5-PC-sparse and d, ml, dl and rdl clusters were E12.5-PC-sparse at E14.5 (preceding section), we considered that lineages of these clusters were also E10.5-PC-sparse or E12.5-PC-sparse in later developmental stages. Therefore, we examined distributions of tdTomato-labeled PCs in PC clusters that were identified by immunostaining of Pcdh10 and EphA4 and referring to our preceding analysis of clusters (Figs. 2–5) in serial coronal sections of TM10.5 and TM12.5 G2A::Ai9 cerebellums at E14.5, E15.5, E16.5 and E17.5 (Fig. 7). E10.5-PC-sparse clusters and E12.5-PC-sparse clusters were identified among all clusters distinguished at each stage (blue and orange areas in panels of Fig. 7*A–D*). This observation indicated that medially- and centrolaterally-located E10.5-PC-sparse clusters belonged to the lineage of the m and c-l clusters (designated as m and c-l lineage clusters), respectively, at E15.5–E17.5 (Fig. 7*B–D*, blue). Similarly, it was indicated that medially-located E12.5-PC-sparse clusters were d lineage clusters, whereas laterally-located E12.5-PC-sparse clusters were either the ml, dl or rdl lineage clusters, respectively (Fig. 7*B–D*, orange). Finally, it was indicated that medially, laterally, and ventrolaterally-located clusters that contained both E10.5-born and E12.5-born PCs were c-m, l, and vl lineage clusters (Fig. 7*B–D*, neither blue or orange), respectively.

The above conclusion was supported by the consistency of the marker expression profile of lineage clusters. The m, d, c-m, c-l, l, vl lineage clusters showed similar marker expression profiles to those of their original E14.5 clusters, although minor changes in the expression profile were sometimes recognized in some cases (Table 4). Among ml, dl, and rdl lineage clusters, which were E12.5-PC-sparse and located next to one another in the lateral cerebellum, the ml cluster at E14.5 and its daughters, or the ml lineage clusters, at later stages consistently showed strong Pcdh10 expression and were identified consequently (lateral orange in Fig. 7*A2, B2, C2, D2*). The dl and rdl lineage clusters were distinguished based on denser E10.5-born PCs and Pcdh10 expression and more rostrolateral positioning of rdl lineage clusters than dl lineage clusters (Table 4). Since their distinction was not necessarily very clear, we sometimes indicate them together by “dl+rdl” in this report.

As a whole, the birthdate-specific labeling of PCs allowed us to identify the lineage of all PC clusters between E14.5 and E17.5 (Table 3).

**Table 3:** Definition of E17.5 PC clusters

Cluster	Definition in Fujita et at. 2012	caudorostral levels	Corl2	Pcdh10	EphA4	FoxP2	Calbindin
va1	va1	13-36%	++	++	+	+++	+
va2-4	va2, va3, va4	6-52%	+++	+	-	++	+
vc1	vc1	6-27%	+++	+++	++	+++	+
vp1-2	vp1, vp2	6-18%	++	+	++	+++	+
vp3-4	vp3, vp4, vc2	6-19%	+++	+	+++	+++	+
vt1	vt1	6-58%	+	++	-	+	++
vt2	vt2	10-58%	+++	+	++	++	-
vt3	vt3	6-58%	++	++	+	+++	+
vt4	vt4	6-58%	+	++	-	+	+
vt5	vt5	10-52%	++	+	-	+	-
ia1-2	ia1, ia2	42-55%	+	+	-	++	-
ia3-5	ia3, ia5	62-71%	++	+	++	+++	-
ia4	ia4, ic4	26-49%	++	+	-	++	-
ic1-2	ic1, ic2	10-26%	+++	+++	+++	++	+
ic3	ic3	23-39%	+	+	+++	+++	-
ip1-2	ip1, ip2	10-23%	++	+	-	++	-
it2	it2, ic5, ip3	23-55%	++	+++	-	++	+
it3	it3	25-80%	++	+	+++	++	-
ha1	ha1	55-77%	++	-	+	++	-
ha2-3	ha2, ha3	83-97%	++	+	++	+++	-
ha4	ha4	77-97%	++	+	+++	++	-
ha5	ha5	77-90%	+++	++	++	++	-
ha6	ha6	80-100%	+	+	+++	++	-
hc1	hc1	52-62%	+	+	-	+	-
hc2	hc2	77-97%	++	-	+	+	-
hp1-2	hp1, hp2	36-68%	+	++	-	+	-
hp3	hp3	71-90%	+	+	-	++	-
hp4	hp4	68-93%	+	+	++	++	-
pf	pf1, pf2	71-90%	+	++	+	++	-
fl1-2	fl1, fl2	49-74%	+	++	-	+	-
fl3	fl3	71-62%	++	++	+	-	-
fl4	fl4	59-100%	+++	++	++	-	+
fl5	fl5	83-100%	++	+	-	-	-
no1-2	no1, no2	6-29%	++	++	++	+	+++
no3	no3	16-24%	++	+	-	+	-
no4	no4	13-21%	+	+	++	+	-
no5	no5	16-20%	+	+	+	+	-

Correspondence to the definition in Fujita et al. (2012), and location of the cluster within the caudorostral level of the cerebellum are shown (columns 2,3). Relative intensity in immunostaining in the present study (columns 4-8) was generally consistent with the previous result (Fujita et al., 2012), but showed some minor differences. In cluster names, “v”, “i”, “h”, “a”, “p”, “t”, “c”, “pf”, “fl”, “no” means vermal, intermediate (paravermis), hemisphere, anterior, posterior, translobular (anterior+posterior), central, parafloccular, floccular, nodular, respectively. The numeral (such as “1” in “vp1-2”) counts the cluster from the medial to the lateral side in each category (“it1” is absent).

At E17.5, the m lineage clusters were located in the most medial area (blue, vt1, vt2, vt3, vc1, vp1-2, in Fig. 7*D4*), whereas the d lineage clusters were located in the laterally neighboring area (orange, va1, vt5, vt4 in Fig. 7*D4*). The location of other lineages of clusters was more complicated. All c-m lineage clusters and some c-l lineage clusters were intermingled and located in the intermediate position lateral to the d lineage clusters (grey, vp3-4, ic1-2, ic3 and light blue, ia1-2, va2-4, ip1-2, in Fig. 7*D4*). More lateral were all ml lineage clusters and some c-l lineage clusters (yellow, it2, ia4 and light blue, it3 in Fig. 7*D4*). All dl+rdl and l lineage clusters and some c-l linage clusters were located in the most lateral position (yellow, hp1-2, hc1, ha2-3, hp4, grey, hc2, ha6, and light blue, ha4 in Fig. 7*D4*). All vl lineage clusters were located in the ventrolateral edge of the E17.5 cerebellum (not shown in Fig. 7*D4*).

The results demonstrated that the positional relationship among clusters changed in some places during the period between E14.5 and E17.5, indicating that the separate migration of divided clusters is one of the bases for the rearrangement of the compartmental organization of the cerebellum. Furthermore, the marker expression profiles of separated clusters that belonged to the same lineage changed differently to some extent in some cases (Table 4).

**Table 4:** Changes in the molecular expression profile in PC clusters from E14.5 to E17.5

E14.5 PC cluster and E17.5 lineage clusters (Definition in Fujita et al. 2012, if it is different)		Changes from E14.5 to E17.5			
		Corl2	Pcdh10	EphA4	FoxP2
m		+ ∼ +++	- ∼ ++	- ∼ ++	+ ∼ +++
	vt1	(+)	(++)	(-)	(+)
	vt2	(+++)	>> +	(++)	(++)
	vc1	(+++)	>> +++	(++)	(+++)
	vp1-2 (vp1, vp2)	(++)	>> +	(++)	(+++)
	vt3	(++)	(++)	(+)	(+++)
	no1-2 (no1, no2)	(++)	(++)	(++)	(+)
d		++	++	–	+++
	vt5		>> +		>> +
	vt4	>> +		>> +	>> +
	va1				
c-m		+++	+ ∼ +++	++	+
	vp3-4 (vp3, vp4, vc2)		(+)	>> +++	>> +++
	ic1-2 (ic1, ic2)		(+++)	>> +++	>> ++
	ic3	>> +	(+)	>> +++	>> +++
	no3	>> ++	>> +	>> -	
	no4	>> +	>> +		
	no5	>> +	>> +	>> +	
c-l		++	+/-	++	++
	va2-4 (va2, va3, va4)	>> +++		>> -	
	ip1-2 (ip1, ip2)			>> -	
	ia1-2 (ia1, ia2)	>> +		>> -	
	it3			>> +++	
	ha4			>> +++	
	ia3-5 (ia3, ia5)				>> +++
ml		+	+++	+++	+++
	it2 (it2, ic5, ip3)	>> ++		>> -	>> ++
	ia4 (ia4, ic4)	> ++	>> +	>> -	>> ++
dl		+++	+/-	++	++
	hp1-2 (hp1, hp2)	>> +	>> ++	>> -	>> +
	hc1	>> +		>> -	>> +
	ha1	>> ++		>> +	
	ha2-3 (ha2, ha3)	>> ++			>> +++
rdl		+++	++	+++	+++
	hp3	>> +	>> +	>> -	>> ++
	hp4	>> +	>> +	>> ++	>> ++
	pf (pf1, pf2)	>> +		>> +	>> ++
l		++	+	++	++
	ha5	>> +++	>> ++		
	ha6	>> +		>> +++	
	hc2		>> -	>> +	>> +
vl		++	+/-	+/-	-
	fl1-2	>> +	>> ++		>> +
	fl3		>> ++		
	fl4	>> +++	>> ++	>> ++	
	fl5				

“∼” indicates the gradient in the molecular expression intensity.

“>>” indicates the change in the molecular expression intensity

### Spatial differentiation of the c-l lineage clusters during the period from E14.5 to E17.5

**Figure 8.**
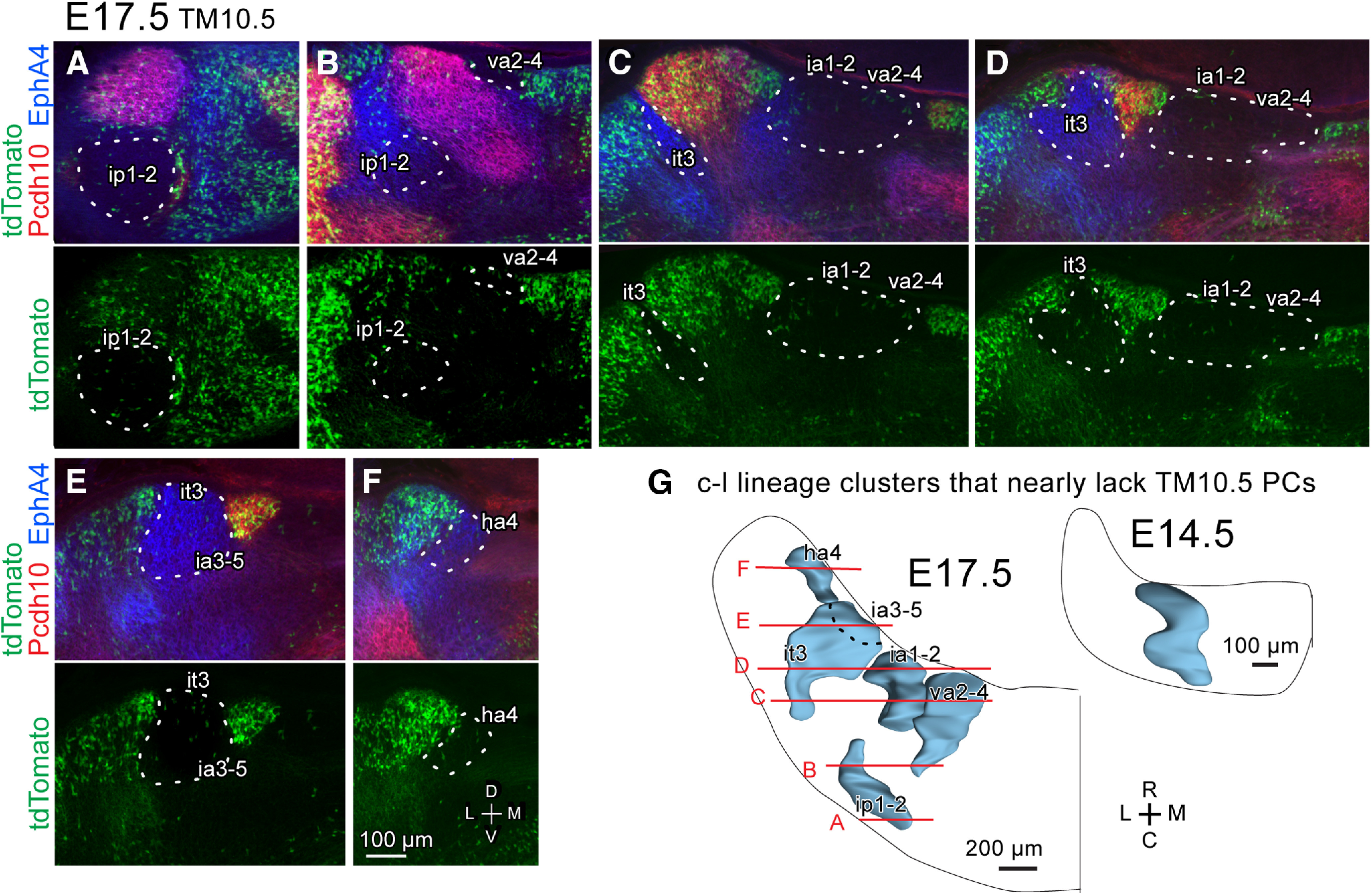
Separated distribution of the E10.5-PC-sparse c-l lineage clusters in the E17.5 cerebellum. ***A–F***, Images of a part of the coronal section at various rostrocaudal levels of the left cerebellum. The top subpanel shows the merged signal of Pcdh10 immunostaining (red), EphA4 immunostaining (blue) and tdTomato expression indicating E10.5-born PCs (green), whereas the bottom subpanel shows only tdTomato expression. White dashed lines demarcate E10.5-PC-sparse areas. Scale bar in ***F*** applies ***A–F***. ***G***, Dorsal view of the 3D scheme of the c-l lineage clusters in the left E17.5 cerebellum, compared with the single c-l cluster in the left E14.5 cerebellum. Black dashed line indicate the contour of the ia3-5 cluster which is located ventral to the it3 cluster. Abbreviations, ha4, ia1-2, ia3-5, it3, ip1-2, va2-4, names of E17.5 clusters; C, caudal; D, dorsal; L, lateral; M, medial; R, rostral.V, ventral.

Among the 37 identified clusters in the E17.5 cerebellum, c-l lineage clusters were located in the most widely-separated areas (light blue, ip1-2, ia1-2, va2-4, it3, ia3-5 and ha4 in Fig. 7*D4*). Although originating from a single c-l cluster, the c-l lineage clusters were located separately at various rostrocaudal and mediolateral positions in the E17.5 cerebellum (Fig. 8*G*). As described in the preceding section, all the c-l lineage clusters were E10.5-PC-sparse, which facilitated their identification, and showed no expression of Pcdh10 (scarce red or green signals in circumscribed clusters in Fig. 8*A–F*) and various expression of EphA4 (blue signals in some circumscribed clusters in Fig. 8*A–F*).

**Figure 9.**
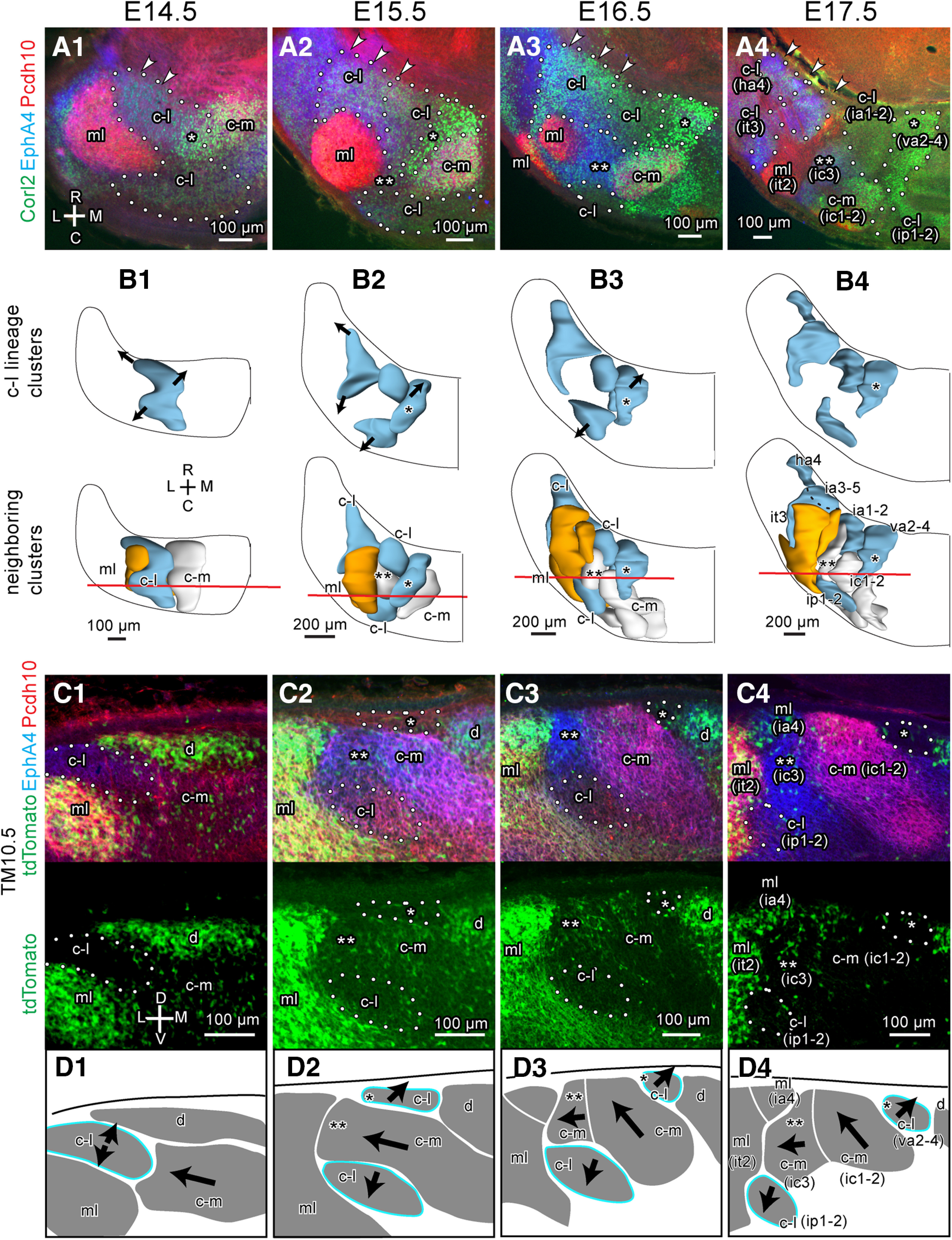
Separation and migration of the c-l lineage cluster from E14.5 to E17.5. ***A***, Horizontal sections of the left paravermal and hemispheric cerebellum at around the central level. Sections were immunostained for Corl2 (green channel), EphA4 (blue channel), and Pcdh10 (red channel). Dashed lines circumscribe c-l lineage clusters. Arrowheads indicate the lateral migration of the rostral c-l lineage clusters. ***B***, Dorsal view of the three-dimensional scheme of reconstructed c-l lineage clusters (blue). In the bottom panel, ml and c-m lineage clusters (orange and white) are added. Red transversal line indicate the position of the coronal section shown in (***B***). Black dashed line indicate the contour of the ia3-5 cluster which is located ventral to the it3 cluster in (***B4***). ***C***, Images of a part of the left cerebellum at the junction between the c-l and c-m lineage clusters in coronal sections. The top image shows merged signals of tdTomato expression, which indicates E10.5-born PCs (green) and immunostaining for EphA4 (blue) and Pcdh10 (red). The bottom subpanel shows only the image of only tdTomato labeling. ***D***, Schematic drawing of clusters shown in ***C***. In ***A–D***, columns of subpanels 1-4 are from E14.5, E15.5, E16.5 and E17.5 cerebellums. Dashed lines indicate c-l lineage clusters that are E10.5-PC-sparse and weak in Pcdh10 expression in ***A*** and ***C***. Single asterisks indicate the most medial part of the c-l cluster or the most medial c-l lineage cluster which had higher expression of Corl2 than the rest of the c-l lineage clusters in ***A–D***. Double asterisks indicate the most lateral part of the c-m cluster or the most lateral c-m lineage cluster which intercalated the c-l lineage clusters in ***A–D***. Arrows indicate the direction of cluster migration. Scale bar in ***C1*** (100 μm) applies to ***C1–C4***. Abbreviations, c-l, c-m, d, ml, names of E14.5 clusters; ha4, ia1-2, ia3-5, ia4, ic1-2, ic3, ip1-2, it2, it3, va2-4, names of E17.5 clusters; C, caudal; D, dorsal; L, lateral; M, medial; R, rostral.V, ventral.

To further clarify the spatial differentiation of c-l lineage clusters during the period between E14.5 and E17.5, we examined positional relationships between c-l lineage clusters and neighboring clusters (Fig. 9). At E14.5, the c-l cluster was located lateral to the c-m cluster and medial to the ml cluster: again, the c-l cluster was identified by the weak Pcdh10 expression, weak Corl2 expression and the lack of E10.5-born PCs (Fig. 9*A1*, Table 2). It occupied the mid-lateral part of the center of the cerebellum with its dorsocaudal part extended laterally to the superficial area dorsal to the ml cluster (Fig. 9*A1, B1*). The rostral part of the c-l cluster was more extended laterally in the position rostroventral to the ml cluster (arrowheads in Fig. 9*A1*). The rostromedial part of the c-l cluster had a slightly stronger Corl2 expression than the rest of the c-l cluster (Fig. 9*A1*, asterisk) and adjoined medially with the c-m cluster which had strong Pcdh10 expression (Fig. 9*C1,D1*).

At E15.5, three major rearrangements occurred in the c-l cluster. Firstly, the most medial part of the c-l lineage cluster was recognized as a separate daughter cluster because of its strong Corl2 expression (single asterisk in Fig. 9*A2, B2, C2, D2*). Secondly, the rostrolateral part of the c-l lineage cluster migrated further laterally in areas rostral and ventral to the ml lineage cluster and increased in EphA4 expression (arrowheads in Fig. 9*A2*). The caudolateral part of the c-l lineage cluster retracted medially. Lastly, the lateral part of the c-m lineage cluster, (Fig. 9*A2, B2*, double asterisk), migrated laterally at the position ventral to the c-l lineage cluster to separate the ventral portion of the c-l lineage cluster into the rostral and caudal parts (Fig. 9*C2, D2*).

At E16.5, the separation of the c-l cluster that started at E15.5 became clearer. The most lateral part of the c-m lineage cluster further migrated laterally to separate the rostral and caudal parts of the c-l lineage cluster completely (Fig. 9*A3, B3, C3, D3*, double asterisk). This lateral migration of the c-m lineage cluster confirmed our previous observation with pcdh10 reporter mice (Vibulyaseck et al., 2017). The rostral part of the c-l lineage cluster was spread widely in the mediolateral direction (arrowheads in Fig. 9*A3, B3*) and subdivided into three daughter subclusters that were recognized by different molecular expression profiles (Fig. 9*A3, B3*). The lateral daughter subcluster migrated laterally and also elongated caudally (blue in Fig 9*B3*).

At E17.5, the separated c-l lineage clusters (ip1-2, ha4, it3, ia1-2, and va2-4 clusters in Fig. 9*A4, B4*) spread in the mediolateral direction at different rostrocaudal levels (arrowheads in Fig. 9*A4*). The laterally migrating Pcdh10-positive c-m cluster (ic1-2 cluster) faced the dorsal surface of the cerebellum (Fig. 9*C4, D4*), as reported previously (Vibulyaseck et al., 2017). The appearance of clear PC-free gaps further firmly separated neighboring daughter clusters (e.g. ia1-2 vs va2-4, in Fig. 9*A4*). Within these daughter clusters, we noticed a developmental change in EphA4 and Corl2 expression; Corl2 expression became much stronger in the va2-4 cluster than other clusters, while EphA4 expression became stronger in the it3 and ha4 clusters than in the va2-4 and ia1-2 clusters. However, Pcdh10 expression remained weak in these c-l lineage daughter clusters throughout the development from E14.5–17.5 (Fig. 9*A4*).

### The lineage of the c-l cluster at adult

**Figure 10.**
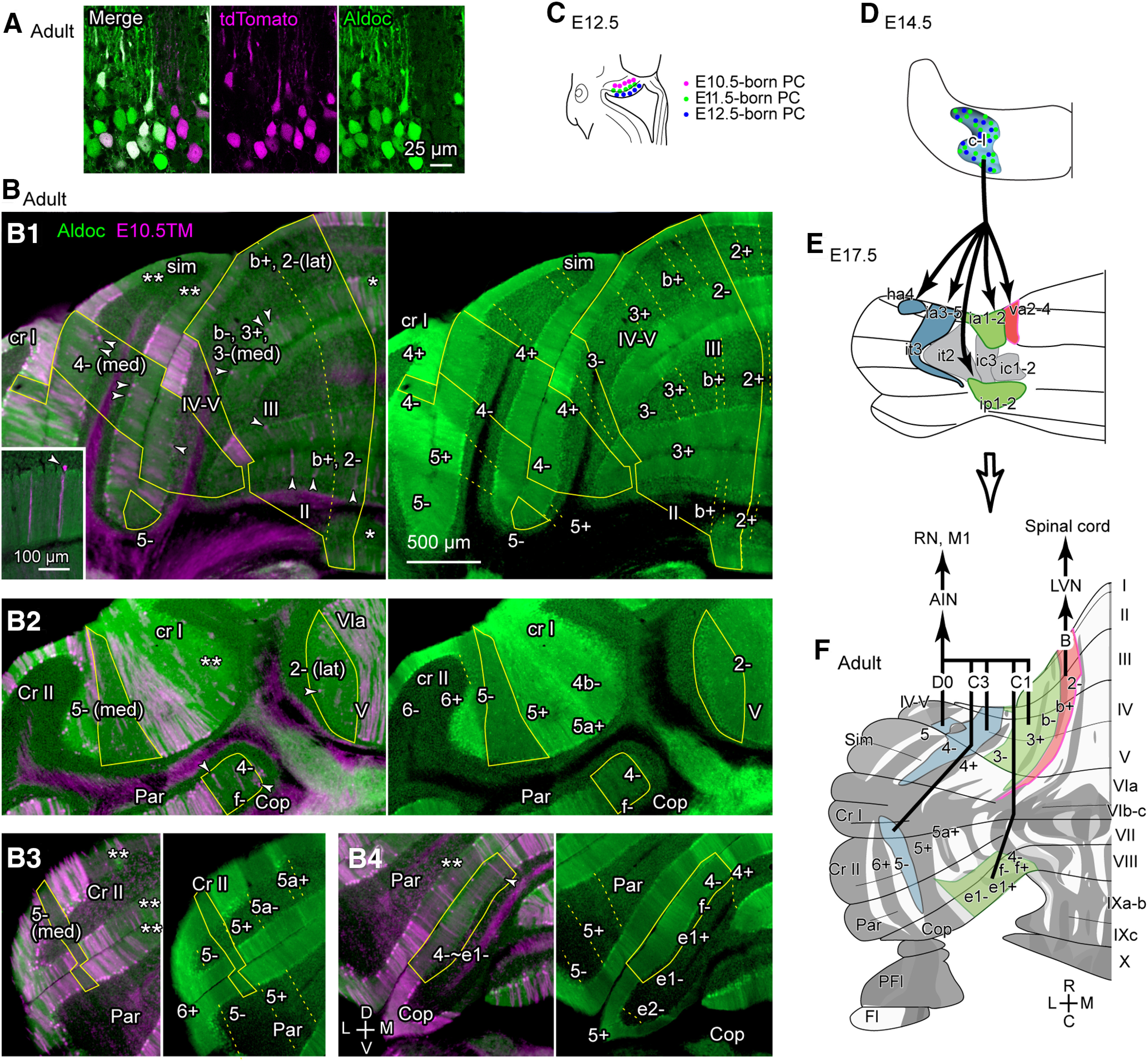
The fate of c-l lineage clusters in the compartmentalization of the adult cerebellar cortex. ***A***, Confocal high magnification images of the cerebellar cortex of the G2A::Ai9::AldocV mouse in which tamoxifen was given at E11.5, showing tdTomato expression in Purkinje cells. ***B***, Four coronal sections of the left cerebellum at rostral (***B1***), central (***B2***) and caudal (***B3, B4***) levels in the G2A::Ai9::AldocV mouse that received tamoxifen at E10.5 and sacrificed at P42. Superimposed images of magenta (tdTomato representing E10.5-born PCs) and green (Venus representing aldolase C expression) channels are shown in the left, while only green channel (Venus, aldolase C) is shown in the right. E10.5-PC-sparse areas that were considered to belong to the lineage of the c-l cluster were circumscribed by yellow lines. Arrowheads indicated labeled PC somata that were observed within the circumscribed area. Zebrin stripes were indicated in the right panels. Scale bar (500 μm) applies to ***B1-B4***. Inset in panel B1 shows sparse Purkinje cell labeling at the dendrite (left) and at the soma and dendrite (arrowhead) in the c-l lineage area. ***C***, Schematic of intermittent PC generation in the ventricular zone of the left embryonic cerebellum. ***D***, Schematic of the c-l cluster composed of randomly located E11.5- and 12.5-born PCs in the left E14.5 cerebellum. ***E***, Schematic of separation and migration of c-l lineage clusters (blue, green and red) at E17.5. Gray clusters are non-c-l-lineage clusters that separate the green clusters into the anterior and posterior parts and separate the blue cluster into the anterior and posterior parts later. ***F***, Mapping of the E10.5-PC-sparse areas that were considered to belong to the lineage of the c-l cluster mapped the unfolded scheme of the entire cerebellar cortex with the zebrin striped pattern (Sarpong et al., 2018). Projections of the B module and C1/C3/D0 modules are shown schematically. Abbreviations: 1+, 1- and so on, aldolase C (zebrin) stripes 1+, 1- and so on; I–X, lobules I–X; a–c, sublobules a–c (as in IXa–b); AIN, anterior interposed nucleus; B, C1, C3, D: modules B, C1, C3 and D0; C, caudal; Cop, copula pyramidis; Cr I, crus I; Cr II, crus II; D, dorsal; Fl, flocculus; ha4, ia1-2, ia3-5, ic1-2,ic3, ip1-2, it2, it3, va2-4 names of E17.5 PC clusters; L, lateral; LVN, lateral vestibular nucleus; M, medial; M1, primary motor cortex; Par, paramedian lobule; PFl, paraflocculus; R, rostral; Sim, simple lobule; V, ventral.

Our previous study (Fujita et al., 2012) has suggested that the E17.5 clusters that were identified as the c-l lineage clusters in the present study (va2-4, ia3-5, ia1-2, it3, ha4, and ip1-2 clusters in the E17.5 cerebellum, Fujita et al., 2012) become zebrin-negative and-lightly-positive stripes in paravermal and hemispheric areas in the anterior and posterior lobules. We tried to confirm the location of c-l lineage stripes directly in the adult cerebellar cortex by using G2A::Ai9::AldocV mice which show tamoxifen-induced birthdate-dependent labeling of neurons with tdTomato as well as labeling of zebrin-positive PCs with Venus fluorescent protein (Fig. 10*A*). We have reported the dependency on tamoxifen injection timing of the general pattern of PC labeling in adult G2A::Ai9::AldocV mice (Zhang et al., 2020). Here, we mapped E10.5-PC-sparse cortical areas by the observation of cerebellar sections of G2A::Ai9::AldocV mice that received tamoxifen at E10.5 (n=2 mice) to identify c-l lineage stripes.

In the adult mouse cerebellar cortex, the striped expression pattern of zebrin (aldolase C) is consistent across individuals and has been identified in detail (Fujita et al., 2014; Sarpong et al., 2018). Accordingly, zebrin stripes are described here by using the common nomenclature (Fujita et al., 2014; Sarpong et al., 2018). Zebrin stripes are mostly designated with a numeral or an alphabet character followed by “+” or “-” (Fig. 1*C*). Note that a pair of stripes in the anterior and posterior lobules, which are named differently, together belongs to the same cerebellar module, because they receive common branching olivocerebellar axons (Sugihara and Shinoda, 2004): for example, a pair of 4+ stripe in anterior lobules and 5+ stripe in posterior lobules form the C2 module and thus considered to be the same or linked stripe, which is designated as “4+//5+”.

In lobules I-V, except for stripe 2+ and the medial part of stripe 2-, most of the paravermal stripes (the lateral part of zebrin stripes 2-, stripes b+, b-, 3+ and 3-) were E10.5-PC-sparse and consequently identified as the fate of c-l lineage clusters (the right circumscribed areas in Fig. 10*B1*). However, the most lateral part of stripe 3- and the entire stripe 4+ contained a high density of E10.5-born PCs. Most of stripe 4- was E10.5-PC-sparse (the left circumscribed area in Fig. 10*B1*). The most lateral part of stripe 4- and entire stripe 5+ contained a high density of E10.5-born PCs. Stripe 5- was E10.5-PC-sparse (the left ventral circumscribed area in Fig. 10*B1*).

In the simple lobule, most of the medial paravermal area had a low-dense distribution of E10.5-born PCs (double asterisks in Fig. 10*B1*). This area was likely to be derived from c-m lineage clusters, which had similarly a low density of E10.5-born PCs in the embryonic cerebellum (Fig. 7*D*). This agrees to the speculation of our previous study that this area originates from E17.5 clusters ic1 and ic2 (Fujita et al., 2012) or cluster ic1-2, which originated from the E14.5 c-m cluster in the present result (Table 2). Zebrin positive stripe 4+ and medially adjacent zebrin negative area contained a high density of E10.5-born PCs. On the contrary, stripe 4-, except for its lateral part, was E10.5-PC-sparse. Stripe 4- was also E10.5-PC-sparse in crus I (the most left circumscribed area in Fig. 10*B1*). These E10.5-PC-sparse areas were identified as the fate of c-l lineage clusters (circumscribed in Fig. 10*B1*) occupied a relatively large portion in the paravermal cerebellar cortex in the rostral cerebellum.

Some tdTomato labeling in the molecular layer inside the circumscribed areas indicated the presence of labeled PC dendrite (left magenta labeling in the inset in Fig. 10*B1*). However, the number of labeled somata that were located within the section (arrowheads in Fig. 10*B1-4*) was rather small (12 PCs in Panel B1, roughly 1-2% against the denominator of all PC somata, ∼700, recognized in the background inside the circumscribed areas), supporting our description “mostly lacking” and “E10.5-PC-sparse”. This percentage (1-2%) was approximately the same as the number of labeled PCs inside the E14.5 c-l cluster (preceding section).

In the rest of cerebellar lobules (lobules VI-X, crus I, crus II, paramedian lobule, copula pyramidis, paraflocculus and flocculus), several areas were E10.5-PC-sparse and identified as the fate of the c-l clusters in the paravermis and hemisphere. These areas included the lateral part of stripe 2- in lobules V-VI (circumscribed in Fig. 10*B2*), the medial part of stripe 5- in crus II (circumscribed in Fig. 10*B2-B3*), and multiple zebrin-negative and faintly zebrin positive stripes in the copula pyramidis and adjacent paramedian lobule (circumscribed in Fig. 10*B2, B4*). Distribution of E10.5-born PCs in the medial paravermal areas in crus I, crus II and paramedian lobule, which are likely to belong to c-m lineage areas (Fujita et al., 2012; Vibryaseck, 2017), appeared a little denser (double asterisks in Fig. 10*B2-4*) than the c-l lineage areas. Other stripes in the paravermal and hemispheric areas had a rather dense distribution of E10.5-born PCs.

The E10.5-PC-sparse zebrin stripes that were considered to belong to the c-l lineage were mapped on the unfolded scheme of the entire cerebellar cortex with the zebrin (aldolase C) striped pattern (Fig. 10*F*; Fujita et al., 2014; Sarpong et al., 2018). The c-l lineage areas were located in zebrin-negative and weakly-positive stripes in the lateral vermis and paravermis in lobules I-V and VIII/copula pyramidis (red and green in Fig. 10*F*), and in the medial hemisphere in lobules I-V, simple lobule, crus II and paramedian lobule (blue in Fig. 10*F*). However, no c-l lineage areas were observed in the central part (lobules VI-VII and apex of crus I) of the cerebellum. Based on the correspondence between the zebrin stripes and cerebellar modules (Sugihara and Shinoda, 2004; Sugihara et al., 2009), the mapped stripes corresponded to the B module (red in Fig. 10*F*), which projects to the lateral vestibular nucleus (Sugihara et al., 2009; Ruigrok et al., 2015), and most of the C1, C3 and D0 modules, which project to the anterior interposed nucleus (green and blue in Fig. 10*F*, Table 5). The results demonstrated that the separation and migration of the c-l cluster during development forms multiple stripes that mainly correspond to the anterior and posterior parts of the C1/C3 module (Fig. 10*C–F*), and also the B module and a part of the D0 module.

**Table 5:** Afferent and efferent connections of the cerebellar modules that were derived from the c-l lineage clusters.

E14.5 PC cluster	Birthdate of PCs	E17.5 PC cluster	Adult zebrin stripe(s)	Module	IO^1^	DCN^2^
c-l	E11.5, E12.5	va2-4	2- (lateral) and b+ in I-V	B	dDAO	LVN
		ip1-2	4-, f+, f-, e1+ in Cop	C1	vDAO	AIN
		ia1-2	b-, 3+ in I-V	C1	vDAO	AIN
		ia3-5 (?)	3-, 3b+, 3b- in III, IV-V	C1	vDAO	AIN
		it3	4-//5- (medial) in III, IV-V, Sim, Cr I, Cr II, Par	C3	vDAO, DM	AIN
		ha4	5- in IV-V	D0	DM	AIN

1 Inferior olive subarea that project to PCs in the stripe (Sarpong et al., 2018; Sugihara and Shinoda, 2004).

2 Cerebellar nucleus subarea that is innervated by PCs in the stripe (Sugihara et al., 2009).

Abbreviations: I-V, lobules I-V; III, lobule III; IV-V, lobule IV-V; AIN, anterior interposed nucleus; Cop, copula pyramidis; Cr I, crus II; Cr II, crus II; DCN, deep cerebellar nucleus; dDAO, dorsal fold of the dorsal accessory olive; DM dorsomedial subnucleus; IO, inferior olive; LVN, lateral vestibular nucleus; Par, paramedian lobule; Sim, simple lobule; vDAO, ventral fold of the dorsal accessory olive.

## Discussion

The present study demonstrated the spatial development of the compartmental organization of the mouse embryonic cerebellum between E14.5 and E17.5. The lineages of the nine E14.5 PC clusters which transformed into 37 clusters at E17.5 were tracked by birthdate-tagging. Furthermore, it was shown that one of the E14.5 clusters named c-l differentiated into several zebrin stripes that belong to the C1/C3 module in the anterior and posterior lobules. The results supported our hypothesis that the rearrangement of embryonic PC clusters shapes the compartmentalization of the adult cerebellar cortex which may underlie its modular organization and the dual somatotopy.

### Cerebellar compartmental organization originate from the differentiation of early PC clusters

The present study revealed a progressive change in the compartmental organization in the embryonic cerebellum. The number of PC clusters recognized by the marker molecule expression profile increased from nine at E14.5 to 37 at E17.5. This increase was accompanied by a significant change in the spatial arrangement of PC clusters between E14.5 and E17.5, as demonstrated in the separation and migration of the c-l lineage clusters in the present study. Since the clustered compartments of PCs in the late embryonic stage (E17.5 in mice) are mostly comparable to the striped compartments of PCs in the adult (Fujita et al., 2012), our clarification of the compartmental differentiation in the period before E17.5 would lead to a better understanding (see below, “Developmental origin of the dual representation of the somatosensorimotor function in the cerebellum”) of adult cerebellar compartmentalization. Compartmental organization at stages earlier than E14.5 was beyond the scope of the present study.

Concerning marker molecules used in this study, we speculate that transcription factors FoxP2 and Corl2 may control the expression of compartment-specific molecules, and adhesion molecule Pcdh10 and receptor tyrosine kinase EphA4 may be involved in cluster formation and cell-to-cell connection between highly-expressing neurons (Vibulyaseck et al., 2017; Sarpong et al, 2018). However, the functional significance of these marker molecules in the development of PC compartmentalization has not been fully clarified. Nevertheless, differences in their expression levels among PCs were useful to detect trackable PC clusters in the present study. Based on different expression intensities of these marker molecules, we distinguished PC clusters in the entire cerebellum in embryonic dates between E14.5 and E17.5 (Figs. 2-5, Table 2)

The PC cluster organization identified in our study conformed to the PC organization that was identified based on gene expression profiling analysis at E14.5 (Wizeman et al., 2019), except for the Nrgn-positive cluster. The Nrgn -positive cluster, which is located widely above the ventricular zone and under other clusters (Wizeman et al., 2019), was not recognized as a cluster in our present study. *Nrgn* mRNA is expressed in neuro-progenitor and immature neurons in cerebral cortical culture (Nazir et al., 2018). Therefore, there is a possibility that Nrgn-positive cluster of Wizeman et al. (2019) represents newly-born PCs that are joining one of the other clusters. Besides, six of our nine E14.5 PC clusters m, c-m, c-l, d, ml, and l corresponded to clusters Ebf, Ebf/Calb1, Nrgn/Calb1, Ebf/Dab1-dorsal, Ebf/Dab1-ventral and Foxp1/Dab1 in Fig. 4*H* of Wizeman et al. (2019), respectively. It would mean that our PC clusters defined by marker molecule expression and PC birthdate and PC clusters defined by gene expression profiling make one-to-one correspondence, except for the Nrgn-positive cluster (above), at the caudal level of the cerebellum. The correspondence of three of our nine E14.5 clusters (dl, rdl and vl) was not known because they were located at the more rostral level than the section shown in Wizeman et al. (2019). As a whole, the high consistency between our E14.5 cluster recognition and PC gene expression profiling support the cluster assembly that we recognized here represents not an arbitrarily defined structure but an essential organization of the E14.5 cerebellum.

### Birthdate-specific neuronal labeling to track the lineage of early PC compartments

Tracing the lineage of early PC clusters in the embryonic cerebellum by birthdate-specific labeling of PCs was the essential method in this study. Since PCs of different birthdates are distributed heterogeneously in different PC compartments (Hashimoto and Mikoshiba, 2003; Namba et al., 2011), birthdate-specific labeling of PCs can be a useful experimental tool to track the lineage of PC clusters. Indeed, the original birthday-specific labeling with the *ascl1* gene CreER-LoxP system showed that PCs labeled by tamoxifen given at E10.5, E11.5 and E12.5 are distributed differently in the adult cerebellum (Sudarov et al., 2011). Such a birthdate-specific labeling system is technically more accessible and appears more sensitive than previously established birthdate-specific labeling methods (systemic injection of 5-bromo-2'-deoxyuridine, BrdU; systemic injection of tritium-labeled thymidine, Altman and Bayer, 1985; or in-utero ventricular injection of replication-defective adenoviral vector, Hashimoto and Mikoshiba, 2004; Namba et al., 2011). G2A mice, in which the *Neurog2* gene is targeted, allowed us to efficiently produce specimens with varying tamoxifen injection dates and survival periods in the present study. The labeling pattern of birthdate-specific PCs in the adult cerebellum in the present study was similar to, but not the same as, that reported with adenoviral vector labeling (Namba et al., 2011). For example, E11.5-born PCs were observed in most of the embryonic PC clusters and the majority of adult zebrin stripes in G2A mice (the present study and Zhang et al., 2020), they were distributed in a smaller number of PC compartments in adenoviral vector labeling. Such discrepancies may be due to some differences in the timing (e.g. different cell cycle points, different durations/efficiencies of labeling activity, etc.) of labeling differentiating neurons between different methods.

### Developmental origin of the dual representation of the somatosensorimotor function in the cerebellum

The birthdate-tagging method employed in the present study revealed that the anteroposteriorly- and mediolaterally-separated stripes belonging to the somatosensorimotor C1/C3 modules originate mostly from the c-l cluster at E14.5, which is composed of E11.5- and E12.5 born PCs (Fig. 10*C-F*). The anteroposterior separation of the C1 module (the medial part of the C1/C3 module) occurred in the medial c-l lineage cluster at E15.5. Besides, the anteroposterior separation of the C3 module (the lateral part of the C1/C3 module) occurs in the early postnatal period in the cluster which originates from the lateral c-l lineage cluster by the lateral migration of the ml lineage cluster (Fig. 10*E*, it3 cluster to be separated anteroposteriorly by ic5/it2 cluster) at P0-P1 as shown previously (Fujita et al., 2012). Although the timing is different, the anatomical process of anteroposterior separation seems similar between these two modules. To further understand this differentiation process, causal mechanisms that induce rostrocaudal separation of c-l lineage clusters are to be studied. The rostro-caudal link has been generally observed in axonal projections in compartments in paravermal and hemispheric modules (Sugihara and Shinoda, 2004; Fujita and Sugihara, 2013). We could not observe evidence of the anteroposterior separation of the B module or the most medial Corl2-strongly positive c-l lineage cluster, as well as evidence of the anteroposterior separation of the D0 module or the most lateral c-l lineage cluster during the embryonic period in the present study, either.

Purkinje cells themselves play an essential role in the formation of the topographic afferent and efferent circuits (Sillitoe et al., 2009, 2010; White et al., 2014). The PC axonal projection and the olivocerebellar projection are directly linked to the compartmental or modular organization of the cerebellar cortex (Sugihara and Shinoda, 2004; Sugihara et al., 2009; Cerminara et al., 2013, Fig. 10F, Table 5). Single climbing fiber axons typically branch rostrocaudally and innervate both the anterior and posterior parts of the same module (Sugihara et al., 2001; Fujita and Sugihara, 2013). Axonal projections of PCs in the anterior and posterior parts of the same module converge on the same small area in the cerebellar nucleus (Sugihara et al., 2009). Such a rostrocaudal relationship in axonal projections is understandable by supposing the same axonal guidance cues (Sillitoe et al., 2009, 2010) expressed by the pair of rostral and caudal PC clusters that originated from the same early cluster. Since PC compartments are topographically connected with subareas of the cerebellar nuclei and inferior olive (Ruigrok et al., 2015), the development of the cerebellar modules may depend on the concurrent development of the compartments in the cerebellar nuclei and inferior olive. Indeed, a genetically-induced defect in the developing cerebellar nuclei produces malformation of the cerebellar cortex (Willett et al., 2019). However, the development of compartments of the cerebellar nuclei or inferior olive has not been clarified yet to the level comparable to the fine compartmentalization shown in the cerebellar cortex (Fujita et al., 2012, 2020).

The mossy fiber projection, which is the main source of afferent axons to the cerebellar cortex, is not as tightly linked to PC compartments as PC axons or olivocerebellar axons (Quy et al., 2011; Biswas et al., 2019; Luo et al. 2020). However, because early mossy fibers initially target PCs (Kalinovsky et al., 2011), the early PC cluster organization may affect the mossy fiber projection pattern. Indeed, single mossy fibers often show branching to the anterior and posterior cerebellums (Biswas et al., 2019), which is similar to the anteroposterior separation of PC clusters shown in the present study. As a whole, the present study showed that the spatiotemporal differentiation process of early PC compartmentalization underlies the anteroposterior dual positioning of somatotopic areas in the cerebellar cortex (Snider, 1950; Stoodley et al., 2012; Guell et al., 2018), one of the most peculiar characteristics of cerebellar functional localization.

The present results propose a hypothesis about the general origin of the cerebellar compartmentalization: adult cerebellar compartments that share similar molecular expression profiles, axonal projections, and functional localization may generally originate from a common early PC compartment in cerebellar development. Besides the C1/C3 modules, our previous finding that the ml cluster at E14.5 became zebrin stripe 4+//5+ (4+ in the anterior cerebellum and 5+ in the posterior cerebellum), or the C2 module (Vibulyaseck et al., 2017) supports this hypothesis. However, the experimental results were not as simple. For example, the most medial part of the c-l cluster showed an increase in expression of Corl2 and was distinguished from the rest of the c-l cluster at E15.5 (Fig. 9*A*). It became stripes 2- and b+, forming the B module, which projects to the lateral vestibular nucleus (Sugihara et al., 2009) for the control of posture and anti-gravity through the lateral vestibulospinal projection (Voogd, 2016). The axonal projection and function of the B module is distinct from those of the C1/C3 module. The B module not only occupies a substantial area in the anterior cerebellum but also exists in the posterior cerebellum, in a small lateral vermal area of lobule VIII (stripe 4-, Fig. 10*B4*; Sugihara et al., 2009). The origin of this caudal B module was not clarified in the present study. The centrolateral part of the c-l cluster formed zebrin stripes 4-//5- (4- in the anterior cerebellum and 5- in the posterior cerebellum, Fig. 10*F*) or the C3 module. The separation of stripe 4-//5- into medial and lateral substripes has been reported in our analysis of the PC birthdates in the adult cerebellar cortex (Zhang et al., 2020). It was noticeable that only the medial substripe of 4-//5- originated from the c-l cluster. Consequently, the lateral substripe of 4-//5- is supposed to originate from a different cluster at E14.5. The most lateral part of the c-l cluster formed a small part of zebrin-negative stripe 5-//6- or the D0 module (Sugihara and Shinoda, 2004). The D0 module is the somatosensorimotor module akin to the C1/C3 modules and containing the area involved in the eye-blinking reflex (Attwell et al., 2001). According to the present results, most parts of the D0 module including the entire caudal parts of the D0 module in the posterior cerebellum, seem to originate from different clusters. Thus, several questions remain regarding functional domains delineated by embryonic clusters and adult striped organization. The relationship between the early PC compartmentalization and the mossy fiber projection pattern is also to be studied.

## Synthesis

Reviewing Editor: Leonard Maler, University of Ottawa Decisions are customarily a result of the Reviewing Editor and the peer reviewers coming together and discussing their recommendations until a consensus is reached. When revisions are invited, a fact-based synthesis statement explaining their decision and outlining what is needed to prepare a revision will be listed below. The following reviewer(s) agreed to reveal their identity: Fredrik Bengtsson. Note: If this manuscript was transferred from JNeurosci and a decision was made to accept the manuscript without peer review, a brief statement to this effect will instead be what is listed below.

Editor comments

Both reviewers agree that this manuscript has very high quality anatomical data. Both reviewers also agree that the manuscript is mostly densely written descriptive morphology and is difficult to follow. And, both reviewers agree that the connections to function are poorly explained.

I have left the reviews almost entirely intact because they are both of high quality and very explicit in what revisions must be undertaken.

Please make all the changes suggested by the reviewers. It is especially important that your Rebuttal contains a complete response to each point raised that notes how the reviewer comments were addressed. This manuscript only requires a final Editorial assessment and I will rely on first carefully reading your rebuttal and then checking in the manuscript as to whether you have responded in a satisfactory manner to each point raised.

I did read the Ms myself and I agree that the data presented are of really high quality. But it is equally important that the description of the data is clear and that the connection to prior published work and functional significance of the data be made clear.

Reviewer #1

In this paper the authors set out to investigate how clusters of Purkinje cells are organized during the development of the cerebellar cortex. In particular the authors claim that they are interested to understand how this organization correlates and underlies the somatotopical and functional organization of the cerebellum.

As in their previous works these authors deliver a meticulous account of the cerebellar anatomy. The paper shows an extensive account of how clusters of Purkinje cells form and transforms during the development of the cerebellum. They use a variety of techniques and as in their previous works the experimental preparation is of very high standard. As far as I can see this part of the paper is impeccable.

However, emphasis of the paper is on somatotopy and functional organization. This is where I have some concerns. Although the word somatotopy is included in the title I can not quite see how the paper relates to somatotopy. There is quite an extensive literature on the somatotopical organization of the cerebellum (nicely reviewed in, A century of cerebellar somatotopy: a debated representation, by Ermanno Manni and Laura Petrosini in Nature Reviews Neuroscience, volume 5, March 2004). The authors need to relate their findings to earlier findings in a more extensive way if it is their aim to clarify functional cerebellar organization and somatotopy if this is their aim.

Firstly, the matter of somatotopic organization in the cerebellum is complex and it has been shown that it can be subject to plasticity (Jörntell and Ekerot 2002). It has been shown for the spinal cord that the functional organization is at least to some part due to sensorimotor feedback from spontaneous movements during development (Peterson et al 2003). The authors need to discuss and relate their findings to such aspects of functional organization.

Secondly, Rosina and Provini 1983, 1987 (J Comp Neurol) show that the same climbing fiber branches provide input to anterior and posterior lobes. Therefore, it is the same neuron in the IO, how does this fit with cell clusters during development? This also needs to be discussed.

I strongly believe that it would be very helpful for the reader to have an initial figure that shows and describes cerebellar somatotopy, cerebellar modules and zebrin banding. As it is now, this is not displayed until in the end of the manuscript (Figure 9).

The initial part of the abstract is a bit hard to follow and I suggest that the authors have another look at it. Maybe something along the lines of: “One of the notable characteristics of the cerebellar cortex is a dual representation of the body (somatotopy) along its anterior-posterior axis. This somatotopy is most clearly seen along the parasagittal cortical strips C1 and C3. C1 and C3 are part of a functional module defined by climbing fiber input from the rostral dorsal accessory olive (rDAO) and their Purkinje Cell projection to anterior interpositus (NIA). In this report, we describe the developmental origin of the C1 and C3 strips.”

There are a few small language errors in the manuscript that needs tending to.

P2 Under the heading significance statement, line 3: apsect-aspects

P18 Line 9: the evidence - remove

Reviewer #2

This paper identifies the developmental clusters of Purkinje cells that become spatially transformed into specific longitudinal pairs of stripes that are linked by climbing fiber inputs, somatotopy, and projection targets in the deep cerebellar nuclei or vestibular nucleus.

This paper examines the spatial pattern of Purkinje cells (PCs) during embryonic development and in adult mice by using a combination of molecular markers which label subsets of PCs and a new birthdating strategy which labels PCs generated on each of several successive days. Although several previous reports from this group and others have already studied PC developmental clusters and their rearrangement into mature clusters and stripes, this paper provides additional information that could be useful for the field, particularly the tight correspondence between specific developmental PC clusters and their mature stripe identity and the demonstration that anterior and posterior PCs in a given stripe originate from the same developmental cluster. The question that they address is: What is the embryonic origin of the odd adult pattern of PCs, in which rostral and caudal PCs in a specific longitudinal stripe exhibit 2 mirror copies of somatotopical organization?

The authors focus specifically on the period between embryonic days 14.5 and 17.5, during which spatially segregated clusters of PCs (which express different combinations of molecules) are transformed from a small number of major clusters (9 in this study, 8 in multiple previous reports from the authors) into a large number of clusters (∼50 in previous report, 37 in this report). The new results demonstrate that the birthdates of PCs in each early cluster are distributed across multiple days, with some clusters comprising very few PCs generated at either the earliest time (E10.5) or the latest time (E13.5). They then identify the ‘lineage’ of PCs from one of the early clusters (“CL”) which can be identified by its lack of PCs generated at E10.5. The results demonstrate convincingly that the embryonic CL cluster matures into several different longitudinal stripes of PCs in the adult, each of which is separated in the central part of the cerebellum by PCs that were generated from a completely different embryonic cluster. Interestingly, the “CL” cluster generates several PC stripes in the ‘C1’ and “C3” zones, which are related to sensory-motor functions of the limbs. But the “CL” cluster also generates PCs in the ‘B’ zone that project to vestibulo-spinal neurons, and also a subset of PCs in the D0 zone, apparently related to eye blink. The main conclusion is that the spatial rearrangement of a specific early cluster of embryonic PCs underlies the cerebellar dual somatotopic organization.

The data are convincing and the figures are generally high quality, but the paper is exceedingly difficult to read and understand in its current form, particularly because the hypotheses and alternatives are not clearly stated. So many visual details are described in the text, with very little helpful framework (hypotheses, questions, if then statements) for understanding what details are specifically important for the reader to understand. The discrepancies between this study and the authors’ previous reports are not clearly explained, and it is difficult to discern exactly what was and was not known prior to this study. Although the Significance Statement was very helpful and nicely written, I could not figure out what the paper was about from the Abstract alone. Similarly, the Introduction and Discussion could make the paper much more accessible to readers (with higher impact) if they guided the reader through alternative hypotheses.

Major comments

1. What is the specific hypothesis, and what are the alternatives?

1a. If the hypothesis is that “The rearrangement of embryonic PC clusters underlies the adult compartmentalization that provides topographic axonal projections and supports functional localization” (as stated in the Abstract and Discussion) what do you mean by ‘functional localization’? What are some alternative hypotheses?

1b. What exactly are you trying to explain with this study? What signals and circuits are responsible for the dual somatotopy that the title focuses on?

Do the PCs encode different regions of the body, or different movements? (if so, please state that so readers understand what you mean). Is the somatotopy because of where PCs project their axons, or where they get their inputs from? Are the climbing fibers or mossy fibers essential for this somatotopy? How does the rearrangement of PCs explain the dual somatotopic representation?

1c. Could alternative hypotheses be that climbing fiber collateralization to anterior and posterior PCs in a single stripe be the basis of the dual organization? Or, that PCs related by their projection pattern and somatopy are born on different days (this was ruled out by the data, but was it formally a possibility prior to this paper?).

1d. Discussion should but does not mention or consider the relevance of the important recent finding that PC development depends on the neurons in cerebellar nucleus that they connect with (Willett et al, 2019 Elife).

2. The abstract last sentence overstates the generality of the findings and the Introduction doesn’t clarify the background required to understand the questions addressed in this study,

2a. The dual somatotopic representation in cerebellum is found throughout the cerebellum, but this paper focuses specifically on the lateral vermis and paravermis. The conclusion sentence of the Introduction is more appropriately restricted to the somatotopy of the paravermis and would be more appropriate to use as a concluding sentence of the abstract.

2b. Please explain some basics of compartmentalization, signficance of stripes, and somatotopy in the introduction of this paper.

2c. The statement in Introduction about previous reports identifying “some 40 clusters separated by PC free gaps” does not seem correct. First, this statement followed by several references that do not discuss the number of developmental clusters or the existence of PC free gaps. Second, the only paper I could find that specified the number of clusters was Fujita et al, 2012, which stated about 50 clusters.

2d. The statement in Introduction that “Each of the E17/5 clusters directly develops into an individual adult PC stripe” includes an irrelevant reference (SIllitoe 2010).

3. The Results indicate discrepancies with the authors’ previous reports that should be accounted for

3a. The subdivision of the previously identified cm cluster into cm and cl clusters raises the possibility that the number of clusters is arbitrarily defined by the specific molecules used to identifiy clusters. What is the functional signficance of slightly higher or lower expression of the molecules that were used to identify and then separate these clusters?

3b. The ∼50 previous clusters identified at E17.5 and P6 by this group have shrunk in this paper to 37 - several previously separated clusters were not distinguished in this paper. Why not? Did the previous report get this fundamental subdivision into 50 clusters wrong? Or, were you unable to identify the previous distinct clusters because you didn’t use a specific molecular marker in this report?

3c. Last sentence of the Results contradicts the previous (2nd to last) sentence. Last sentence appears to be motivated by desire to make a simple story about C1/C3 module development, while 2nd to last sentence appears to be an accurate summary of the results”.

"The separation of the c-l cluster...mainly correspond to anterior and posterior parts of the C1/C3 module"

vs

"B module... and most of the C1, C3, and D0 modules"

Specific Comments

1. Terminology for cluster names should be defined in this paper: eg It3, va4, hp3

2. The figures with small insets showing schematics of cluster subdivisions are difficult to see clearly because some of the figure is obscured by the schematic, which is mostly too small to compare directly with the figure. The figures were more clear in previous publications in which the authors used dotted lines to demarcate subdivisions, or showed the colored figures with real data next to the same size schematics.

Also, please label the deep cerebellar nuclei.

3. Please check Tables carefully for accuracy and mutual consistency. For examples:

- Table 1: no reference for the anti-rat Igg

- Table 2: va1-2 is listed under ‘D’, but va2-4 is listed under ‘c-l’. There is no it1 in this table

- Table 3: 0-100% of what?

- Table 2 lists ic3 under ‘CM’ but, but the same ic3 is listed under “CL” in Table 4

4. Specific words used multiple times in confusing ways:

- “then” (p14, 9 lines from bottom; p17; 14 lines from bottom; p18, 6 lines from bottom"

Because ‘then’ has temporal associations, I got confused reading the above sentences because the term “then” was used instead of “thus", or “it follows”.

- “nearly lacking", “near lacked”. (at least 12 useages throughout the text)

This is an awkward phrase that is difficult to parse. “nearly” makes it more confusing because near implies physical proximity. ‘Mostly lacked’ would be better, but if the authors could just establish explicility that these regions are predominantly devoid of PCs born on specific days, they could use a simpler term, eg “E10.5 PC sparse region”.

5. pcdh10 is not defined in the Abstract, nor is its relevance or the specific focus on the ‘c-l’ cluster made clear.

6. Abstract line 6-8 is not clear, especially “that supports the topographic connections and supports the functional localization”. What does this mean? (what does it mean to ‘provide a topographic connection’ or to ‘support a functional localization’?

7. Significance statement is very clearly written. Is the 2nd line from the end an overstatement? This paper focused on the CL cluster, not “all of the clusters in later stages”.

8. Introduction: The statement that the C1/C3 module underlies the anterior-posterior representation of dual somatosensorymotor function seems overgeneralized. Don’t the vermis and hemispheres also have this dual somatotopy? Please clarify whether this is restricted to C1/C3.

9. Methods: Please define “an appropriate combination” of pseudocolor - what is appropriate?

10. Results line 3: “developmental transformation has been hardly clarified”. ‘hardly’ is not easy to understand. Presumably this means ‘few studies have focused on the developmental transformation’. But, what about all of the papers previously published by the authors? Please clarfiy what has and has not been clarified in prior studies so we can understand the specific questions addressed in this one.

11. Results page 9, end of first section. The discrepancy with previous work from this lab raises a concern about the conclusions from this and previous studies - which ones are accurate? How many clusters are there 50, 37, or some arbitrary number based on molecular expression?

12. Results birthdate section - please refer to the corresponding Table here

13. Results p 11, line 4. Doesn’t the inclusion of 2 or more birthdates in each cluster make it more difficult (not easier) to track clusters using birthdating? Perhaps you mean that the exclusion of specific birthdates in some clusters made it easier to distinguish them from neighboring clusters.

14. Results p 11, last line: E17.5 is used redundantly here

15. Results p 14, 3 lines from end: “...PCs, which extend the dendritic arbor....”. This is confusing

16. Results p 15, paragraph “in the central...” - what is the point of this paragraph?

17. Discussion, p17 -6-8 lines from bottom: Too many useages of “or” in this sentence - please divide into more sentences.

17. Discussion p17 last lines - please comment on role of deep cerebellar nucleus in PC development

18. Please implications of climbing fiber collaterals to anterior and posterior regions that share developmental origin.

19. Discussion p18: “dual representation of somatosensorymotor function”. What do you mean by function here?

20. Discussion p18 “ The present results propose a hypothesis...” Please state the alternative hypotheses, and clarify whether your results are consistent with your hypothesis or not.

The end of this paragraphs contains several caveats that seem to require some expertise to understand. Why the use of “however” for the D0 module statement?

## Author Response

Dear Dr. Maler,

Thank you very much for handling our manuscript and for your reply. We are very happy to hear that you and the reviewers came to the positive and constructive decision about our manuscript. We thank the reviewers for useful and insightful comments. We addressed each of the reviewer’s points to improve the manuscript as explained below.

The use of estimation statistics does not apply to our manuscript, since it is a descriptive study without any intervention.

We respectfully submit a revised version of the manuscript. As you indicated, we have provided a clean copy of the revised manuscript as well together with the one in which our changes are indicated in colors.

Below we highlight major changes that have been made:

(1)Substantial portions of the Abstract, Introduction, Results, and Discussion have been rewritten thoroughly to improve readability. Our test hypotheses have been clearly described. We also described a better introduction of the cerebellar somatotopy than in the previous manuscript. We have replaced a sentence with a phrase “nearly lacked E10.5-born PCs” with a sentence with an adjective “E10.5-PC-sparse” in many places according to Reviewer #2, which made our description simpler in many places.

(2)One new figure (Fig. 1) has been added to improve our introduction of cerebellar somatotopy, modules, aldolase C (zebrin) stripes, and compartmental development. The tables are also revised in format.

(3)The apparent discrepancy between the present finding and our previous results in the previous manuscript is not a discrepancy but rather a technically different way of recognizing patterns. We revised the text to explain why the recognized number of E17.5 clusters is different from the number in the previous paper and to eliminate the concern about this point.

(4)Although the editor and reviewers did not mention the English of our writing, we checked the English of our new manuscript thoroughly and also asked our colleague from an English-speaking country to double-check the English.

In the pages below, please find the responses to the reviewers’ comments.

Best regards,

Izumi Sugihara, M.D., Ph. D.

Department of Systems Neurophysiology

Tokyo Medical and Dental University Graduate School of Medical and Dental Sciences

Center for Brain Integration Research

1-5-45 Yushima

Bunkyo-ku, Tokyo 113-8519, Japan

Phone: 81(Japan)-3-5803-5152

Fax: 81(Japan)-3-5803-5155

e-mail: isugihara.phy1@tmd.ac.jp

http://www.tmd.ac.jp/med/eng/eng/phy1-E.html

*********************************************************************************

-

Editor comments:

Both reviewers agree that this manuscript has very high quality anatomical data. Both reviewers also agree that the manuscript is mostly densely written descriptive morphology and is difficult to follow. And, both reviewers agree that the connections to function are poorly explained.

I have left the reviews almost entirely intact because they are both of high quality and very explicit in what revisions must be undertaken.

Please make all the changes suggested by the reviewers. It is especially important that your Rebuttal contains a complete response to each point raised that notes how the reviewer comments were addressed. This manuscript only requires a final Editorial assessment and I will rely on first carefully reading your rebuttal and then checking in the manuscript as to whether you have responded in a satisfactory manner to each point raised.

I did read the Ms myself and I agree that the data presented are of really high quality. But it is equally important that the description of the data is clear and that the connection to prior published work and functional significance of the data be made clear.

Response: We have revised the manuscript according to all comments of reviewers. Our response is written below each of the reviewers’ comments. The relationship to our prior work and functional significance of the data has been clearly made.

Reviewer #1

In this paper the authors set out to investigate how clusters of Purkinje cells are organized during the development of the cerebellar cortex. In particular the authors claim that they are interested to understand how this organization correlates and underlies the somatotopical and functional organization of the cerebellum.

As in their previous works these authors deliver a meticulous account of the cerebellar anatomy. The paper shows an extensive account of how clusters of Purkinje cells form and transforms during the development of the cerebellum. They use a variety of techniques and as in their previous works the experimental preparation is of very high standard. As far as I can see this part of the paper is impeccable.

However, emphasis of the paper is on somatotopy and functional organization. This is where I have some concerns. Although the word somatotopy is included in the title I can not quite see how the paper relates to somatotopy. There is quite an extensive literature on the somatotopical organization of the cerebellum (nicely reviewed in, A century of cerebellar somatotopy: a debated representation, by Ermanno Manni and Laura Petrosini in Nature Reviews Neuroscience, volume 5, March 2004). The authors need to relate their findings to earlier findings in a more extensive way if it is their aim to clarify functional cerebellar organization and somatotopy if this is their aim.

Response: In response to this comment, we revised the manuscript in three aspects. 1) Since our study is purely anatomical without physiological identification of somatotopy, we have refrained from writing that this study revealed something about the “somaotopy” or “somatotopic representation” or functional localization”. Instead we have written that we revealed something about the “somatotopic areas” or “C1/C3 module”. 2) We also improved the description in the Abstract and Introduction to clearly state our hypothesis and aim of the study. In the description of the hypothesis and aim, we refrained from overstating by restricting the description within the anatomical aspect. 3) We have improved the Introduction and the initial introductory part of the Abstract to introduce the relationship between the cerebellar somatotopic representation and cerebellar compartments, better than the previous manuscript, within the word number limits of these sections. We have cited the review by Ermanno Manni and Laura Petrosini (2004, Nature Neurosci).

Our revisions to your itemized comments (below), as a whole, improved the manuscript in these directions.

Firstly, the matter of somatotopic organization in the cerebellum is complex and it has been shown that it can be subject to plasticity (Jörntell and Ekerot 2002). It has been shown for the spinal cord that the functional organization is at least to some part due to sensorimotor feedback from spontaneous movements during development (Peterson et al 2003). The authors need to discuss and relate their findings to such aspects of functional organization.

Response: Jörntell and Ekerot (2002) examined the plasticity of neuronal responses in C3 modules in the cat. (We could not identify Peterson et al 2003, unfortunately.) Thus, activity-dependent plasticity indicated by the Reviewer occurs mainly at the cellular level. However, we deal with the anatomical structure formed by a big population of cells. Although such cellular and populational events, both of which contribute to the formation of somatotopy, cannot be fully distinguished, the present study deals with the populational anatomical aspect. To include the contribution of activity-dependent plasticity to compartmentalization, we added a brief sentence at the end of the first paragraph of the Introduction: “Activity-dependent plastic tuning of neuronal responses occurs within the anatomical frame of cerebellar modules (Jörntell and Ekerot, 2002).” in the middle of our revision work. However, while working with other parts and aspects of the Introduction in response to other comments of reviewers, we had to omit this phrase mainly because of the word number limit of the Introduction.

Secondly, Rosina and Provini 1983, 1987 (J Comp Neurol) show that the same climbing fiber branches provide input to anterior and posterior lobes. Therefore, it is the same neuron in the IO, how does this fit with cell clusters during development? This also needs to be discussed.

Response: We appreciate this comment that indicates the importance of the present study. In the original manuscript, we wrote just one sentence concerning this. “The PC axonal projection and the olivocerebellar projection are directly linked to the PC compartmental or cortical modules (Sugihara et al., 2004; Sugihara et al., 2009; Cerminara et al., 2013, Fig. 9F, Table 5).” Here, we have expanded and clarified this discussion about rostrocaudal projections by adding a few sentences. “Single climbing fiber axons typically branch rostrocaudally and innervate both the anterior and posterior parts of the same module (Sugihara et al., 2001; Fujita and Sugihara, 2013). Axonal projections of PCs in the anterior and posterior parts of the same module converge on the same small area in the cerebellar nucleus (Sugihara et al., 2009). Such a rostrocaudal relationship in axonal projections is understandable by supposing the same axonal guidance cues (Sillitoe et al., 2009, 2010) expressed by the pair of rostral and caudal PC clusters that originated from the same early cluster.” We cited our papers instead of those by Rosina and Provini works (1983, 1987) since ours show the general rostrocaudal relationship of climbing fiber projection at the level of single axons. (Discussion, page 19, line 668-677).

I strongly believe that it would be very helpful for the reader to have an initial figure that shows and describes cerebellar somatotopy, cerebellar modules and zebrin banding. As it is now, this is not displayed until in the end of the manuscript (Figure 9).

Response: We have added new Figure 1, in which we showed (1) cerebellar somatotopy, (2) cerebellar modules, (3) aldolase C compartments (4) postnatal immature stripes, and (5) embryonic clusters, schematically on the unfolded scheme of the mouse cerebellar cortex. We think this would be a good introductory figure for readers. The previous Figure 1 is now named Figure 2, and so on.

The initial part of the abstract is a bit hard to follow and I suggest that the authors have another look at it. Maybe something along the lines of: “One of the notable characteristics of the cerebellar cortex is a dual representation of the body (somatotopy) along its anterior-posterior axis. This somatotopy is most clearly seen along the parasagittal cortical strips C1 and C3. C1 and C3 are part of a functional module defined by climbing fiber input from the rostral dorsal accessory olive (rDAO) and their Purkinje Cell projection to anterior interpositus (NIA). In this report, we describe the developmental origin of the C1 and C3 strips.”

Response: We have revised the Abstract significantly. The initial part was re-written by considering the reviewer’s comment, although we could not incorporate wholly the reviewer’s writing because of word number limitation. We think the new Abstract is much easier to read for readers.

There are a few small language errors in the manuscript that needs tending to.

P2 Under the heading significance statement, line 3: apsect-aspects

P18 Line 9: the evidence - remove

Response: We have revised these writing errors.

Reviewer #2

This paper identifies the developmental clusters of Purkinje cells that become spatially transformed into specific longitudinal pairs of stripes that are linked by climbing fiber inputs, somatotopy, and projection targets in the deep cerebellar nuclei or vestibular nucleus.

This paper examines the spatial pattern of Purkinje cells (PCs) during embryonic development and in adult mice by using a combination of molecular markers which label subsets of PCs and a new birthdating strategy which labels PCs generated on each of several successive days. Although several previous reports from this group and others have already studied PC developmental clusters and their rearrangement into mature clusters and stripes, this paper provides additional information that could be useful for the field, particularly the tight correspondence between specific developmental PC clusters and their mature stripe identity and the demonstration that anterior and posterior PCs in a given stripe originate from the same developmental cluster. The question that they address is: What is the embryonic origin of the odd adult pattern of PCs, in which rostral and caudal PCs in a specific longitudinal stripe exhibit 2 mirror copies of somatotopical organization?

The authors focus specifically on the period between embryonic days 14.5 and 17.5, during which spatially segregated clusters of PCs (which express different combinations of molecules) are transformed from a small number of major clusters (9 in this study, 8 in multiple previous reports from the authors) into a large number of clusters (∼50 in previous report, 37 in this report). The new results demonstrate that the birthdates of PCs in each early cluster are distributed across multiple days, with some clusters comprising very few PCs generated at either the earliest time (E10.5) or the latest time (E13.5). They then identify the ‘lineage’ of PCs from one of the early clusters (“CL”) which can be identified by its lack of PCs generated at E10.5. The results demonstrate convincingly that the embryonic CL cluster matures into several different longitudinal stripes of PCs in the adult, each of which is separated in the central part of the cerebellum by PCs that were generated from a completely different embryonic cluster. Interestingly, the “CL” cluster generates several PC stripes in the ‘C1’ and “C3” zones, which are related to sensory-motor functions of the limbs. But the “CL” cluster also generates PCs in the ‘B’ zone that project to vestibulo-spinal neurons, and also a subset of PCs in the D0 zone, apparently related to eye blink. The main conclusion is that the spatial rearrangement of a specific early cluster of embryonic PCs underlies the cerebellar dual somatotopic organization.

The data are convincing and the figures are generally high quality, but the paper is exceedingly difficult to read and understand in its current form, particularly because the hypotheses and alternatives are not clearly stated. So many visual details are described in the text, with very little helpful framework (hypotheses, questions, if then statements) for understanding what details are specifically important for the reader to understand. The discrepancies between this study and the authors’ previous reports are not clearly explained, and it is difficult to discern exactly what was and was not known prior to this study. Although the Significance Statement was very helpful and nicely written, I could not figure out what the paper was about from the Abstract alone. Similarly, the Introduction and Discussion could make the paper much more accessible to readers (with higher impact) if they guided the reader through alternative hypotheses.

Response: We clarified the hypothesis and revised the manuscript (and figures) according to all the comments by this reviewer. Consequently, the readability of the manuscript has been significantly improved, we believe.

Major comments

1.What is the specific hypothesis, and what are the alternatives?

Response: In the revised manuscript, we have improved the hypothesis description but did not add the alternative hypothesis description. Please look at our response to 1a-1d (below).

1a. If the hypothesis is that “The rearrangement of embryonic PC clusters underlies the adult compartmentalization that provides topographic axonal projections and supports functional localization” (as stated in the Abstract and Discussion) what do you mean by ‘functional localization’? What are some alternative hypotheses?

Response: In the revised manuscript, we have revised the hypothesis writing. We omitted “functional localization” to be focused only on the anatomical aspect: “we hypothesized that the rearrangement of embryonic PC clusters shapes the adult cerebellar compartmentalization “ (Page 2, line 46-47).

"Therefore, we hypothesized that the rearrangement of embryonic PC clusters is essential in shaping the compartmental organization of the adult cerebellar cortex which includes its modular organization and the dual somatotopic areas” (Introduction, page 4, line 113-115).

"The results supported our hypothesis that the rearrangement of embryonic PC clusters shapes the compartmentalization of the adult cerebellum which may underlie its modular organization and the dual somatotopy.” (Discussion, page 17, line 574-576). In the middle of the revision process, we wrote the alternative hypothesis accordingly. However, we have eventually omitted the alternative hypothesis description, since our study is a descriptive study of the normal anatomy and development and it does not employ any intervention procedures. The alternative hypothesis description seemed to complicate the contents of the Introduction (and Discussion) rather than improving readability and comprehension. Even though we have not written the alternative hypothesis, we think we have shown that the results support our hypothesis well in the revised manuscript.

We added an introductory figure (New Figure 1) according to the comments of the other reviewer. This Figure may also help to clarify our hypothesis by putting the somatotopy scheme (Fig. 1A) and compartment development schemes (Fig. 1C-E) together.

1b. What exactly are you trying to explain with this study? What signals and circuits are responsible for the dual somatotopy that the title focuses on?

Do the PCs encode different regions of the body, or different movements? (if so, please state that so readers understand what you mean). Is the somatotopy because of where PCs project their axons, or where they get their inputs from? Are the climbing fibers or mossy fibers essential for this somatotopy? How does the rearrangement of PCs explain the dual somatotopic representation?

Response: The dual representation of somatotopy (or dual somatotopy) in the cerebellum has been long described in textbooks. The separate rostrocaudal localization of the somatotopy in the paravermal area is the essential point of the dual somatotopy which is dealt with in our manuscript. One of the underlying anatomical mechanisms is the rostrocaudal separate arrangement of the C1/C3 module, which are innervated by rostrocaudally branching climbing fibers (Sugihara and Shinoda, 2004; Fujita and Sugihara, 2013). This study is focused on the developmental origin of the C1/C3 module.

As the reviewer indicates in his comment, many things of different aspects are involved in the somatotopy, PC encoding, climbing fiber inputs, and mossy fiber inputs. Some aspects of underlying mechanisms may be simplified or not fully described in the present manuscript because of the limitation of the word number.

According to the reviewers’ comments, we have rewritten the Abstract and Introduction with significant improvement. The description of the somatotopy has been improved and underlying mechanisms have been written as much as possible within the length limit of these sections. Therefore, we hope the question the reviewer raises “How does the rearrangement of PCs explain the dual somatotopic representation?” is better understood in the revised manuscript. Please look at the initial part of the Abstract and the first and next paragraphs of the Introduction.

1c. Could alternative hypotheses be that climbing fiber collateralization to anterior and posterior PCs in a single stripe be the basis of the dual organization? Or, that PCs related by their projection pattern and somatopy are born on different days (this was ruled out by the data, but was it formally a possibility prior to this paper?).

Response: The alternative hypothesis would be: “the rearrangement of embryonic PC clusters has no direct relationship with the adult cerebellar compartmentalization has not yet been excluded.” or “the anterior and posterior somatotopic areas originate from distinct early PC clusters.” While revising the manuscript, we thought that writing the alternative hypothesis would not improve readability or comprehension of the manuscript. Instead, it needed some number of words. Therefore, we have decided not to write the alternative hypothesis. Although our writing is different from what the reviewer proposes, we think the revised hypothesis made the manuscript more readable and easier to understand.

"We hypothesized that the rearrangement of embryonic PC clusters shapes the adult cerebellar compartmentalization “ (Page 2, line 46-47).

"Therefore, we hypothesized that the rearrangement of embryonic PC clusters is essential in shaping the compartmental organization of the adult cerebellar cortex which includes its modular organization and the dual somatotopic areas” (Introduction, page 4, line 113-115).

1d. Discussion should but does not mention or consider the relevance of the important recent finding that PC development depends on the neurons in cerebellar nucleus that they connect with (Willett et al, 2019 Elife).

Response: We have added some sentences to discuss the relevance of the cerebellar nuclei development: “Since PC compartments are topographically connected with subareas of the cerebellar nuclei and inferior olive (Ruigrok et al., 2015), the development of the cerebellar modules may depend on the mutual development of the compartments in the cerebellar cortex, cerebellar nuclei, and inferior olive. Indeed, a genetically-induced defect in the developing cerebellar nuclei produces malformation of the cerebellar cortex (Willett et al., 2019). However, the development of compartmentalization of the cerebellar nuclei or inferior olive has not been clarified yet to the level comparable to the fine compartmentalization shown in the cerebellar cortex (Fujita et al., 2012; Fujita et al., 2020)” in the revised manuscript (Discussion, page 20, line 677-685).

2. The abstract last sentence overstates the generality of the findings and the Introduction doesn’t clarify the background required to understand the questions addressed in this study,

Response: We have rewritten and thoroughly clarified the last sentence of the Abstract and the Introduction. Please look at our responses to 2a-2d (below).

2a. The dual somatotopic representation in cerebellum is found throughout the cerebellum, but this paper focuses specifically on the lateral vermis and paravermis. The conclusion sentence of the Introduction is more appropriately restricted to the somatotopy of the paravermis and would be more appropriate to use as a concluding sentence of the abstract.

Response: We have rewritten the last sentence of the Abstract: “The results indicate that the spatial rearrangement of embryonic PC clusters is involved in forming the dual somatotopic areas in the adult mouse paravermal cerebellar cortex.” (Abstract, page 2, line 57-59) to restrict the conclusion to C1/C3 module in the paravermal area The conclusion sentence of the Introduction in the original manuscript has been omitted.

2b. Please explain some basics of compartmentalization, signficance of stripes, and somatotopy in the introduction of this paper.

Response: Similar comments were also given by Reviewer #1. We have rewritten the Introduction within the word number limit (750 words). We have added a general introduction of somatotopy and citation of newly created Figure 1 (Introduction, 1st paragraph). The functional and anatomical significance of stripes and compartmentalization was added in the second paragraph. This Figure may also help to clarify our hypothesis by putting the somatotopy scheme (Fig. 1A) and compartment development schemes (Fig. 1C-E) together.

2c. The statement in Introduction about previous reports identifying “some 40 clusters separated by PC free gaps” does not seem correct. First, this statement followed by several references that do not discuss the number of developmental clusters or the existence of PC free gaps. Second, the only paper I could find that specified the number of clusters was Fujita et al, 2012, which stated about 50 clusters.

Response: We appreciate this comment which indicated our mistake. We described 54 clusters in Fujita et al. 2012. We have changed “some 40” to “some 50” in the revised manuscript.

2d. The statement in Introduction that “Each of the E17/5 clusters directly develops into an individual adult PC stripe” includes an irrelevant reference (SIllitoe 2010).

Response: Sillitoe et al (2010) has been removed from this sentence in the revised manuscript (Introduction, page 3, line 111-112).

3. The Results indicate discrepancies with the authors’ previous reports that should be accounted for

Response: The discrepancies between the descriptions in our previous reports and this manuscript, which the reviewer noticed, were produced partly because the first author is the new member of the lab. In the revised manuscript, the authors who were involved in previous studies (Hirofumi Fujita and Izumi Sugihara) have checked details many times to get rid of such discrepancies. Please look at our response to 3a-3c (below).

3a. The subdivision of the previously identified cm cluster into cm and cl clusters raises the possibility that the number of clusters is arbitrarily defined by the specific molecules used to identifiy clusters. What is the functional signficance of slightly higher or lower expression of the molecules that were used to identify and then separate these clusters?

Response: We have added sentences to describe the possible functional significance of higher (and lower) expression of these molecules: “Concerning marker molecules used in this study, we speculate that transcription factors FoxP2 and Corl2 may control the expression of compartment-specific molecules, and adhesion molecule Pcdh10 and receptor tyrosine kinase EphA4 may be involved in cluster formation and cell-to-cell connection between highly-expressing neurons (Vibulyaseck et al., 2017; Sarpong et al, 2018), the functional significance of these marker molecules in the development of PC compartmentalization has not been fully clarified. Nevertheless, differences in their expression levels among PCs were useful to detect trackable PC clusters in the present study.” (Discussion, page 17, line 592-601).

In the Discussion, we have emphasized the correspondence between our nine clusters and the PC organization identified based on gene expression profiling analysis at E14.5 (Wizeman et al., 2019) (Page 17, paragraph 3, line 602-620). At E17.5, the number of recognized clusters may be dependent on how to recognize cluster separation (Results, page 9, the last paragraph).

3b. The ∼50 previous clusters identified at E17.5 and P6 by this group have shrunk in this paper to 37 - several previously separated clusters were not distinguished in this paper. Why not? Did the previous report get this fundamental subdivision into 50 clusters wrong? Or, were you unable to identify the previous distinct clusters because you didn’t use a specific molecular marker in this report?

Response: We recognized the same clusters as described by Fujita et a. 2012. But, decided to combine some of them to facilitate analysis and simplify description. We have rewritten sentences to make this point clear: “Although E17.5 cerebellum contains 54 clusters identified with detailed analyses (Fujita et al., 2012), this study focused on the more qualitative distinction of clusters to facilitate analyses and simplify description. Namely, neighboring clusters that had only slightly different molecular expression profiles and/or not clearly separated from one another by intercalating PC-free gaps were combined. For example, our cluster vp1-2 includes clusters vp1 and vp2 of Fujita et al. (2012). We combined 11 sets of two or three neighboring E17.5 clusters into single clusters, resulting in a total of 37 clusters in place of 54. We adopted the nomenclature (Table 3) from Fujita et al. (2012) to designate F17.5 clusters in the present study.” (Results, page 9, line 307-315).

3c. Last sentence of the Results contradicts the previous (2nd to last) sentence. Last sentence appears to be motivated by desire to make a simple story about C1/C3 module development, while 2nd to last sentence appears to be an accurate summary of the results”.

"The separation of the c-l cluster...mainly correspond to anterior and posterior parts of the C1/C3 module"

vs

"B module... and most of the C1, C3, and D0 modules"

Response: We have rewritten the last sentence of Results: “The results demonstrated that the separation and migration of the c-l cluster during development forms multiple stripes that mainly correspond to the anterior and posterior parts of the C1/C3 module (Fig. 10C-F), and also the B module and a part of the D0 module.”(Results, page 16, line 563-566).

Specific Comments

1. Terminology for cluster names should be defined in this paper: eg It3, va4, hp3

Response: Terminology of Fujita et al., 2012 has been explained in the footnote of Table 3 “In the terminology of Fujita et al., 2012, “v", “i", “h", “a", “p", “t", “c", “fl", “no” means vermal, intermediate (paravermis), hemisphere, anterior, posterior, translobular (anterior+posterior), central, floccular, nodular, respectively. The numeral (such as “1” in “vp1”) counts the cluster from the medial to the lateral side in each category (“it1” is absent).” (Footnote of Table 3).

2. The figures with small insets showing schematics of cluster subdivisions are difficult to see clearly because some of the figure is obscured by the schematic, which is mostly too small to compare directly with the figure. The figures were more clear in previous publications in which the authors used dotted lines to demarcate subdivisions, or showed the colored figures with real data next to the same size schematics.

Also, please label the deep cerebellar nuclei.

Response: When we submitted this manuscript to a different journal before, we drew white dotted lines to indicate the boundary between clusters in Figures 1-4. One reviewer gave us a comment that the dotted lines hid information of the image. Therefore, we omitted dotted lines and added schemes to indicate cluster identification in this submission to eNeuro. Here we have re-introduced dotted lines to the suggestion of the reviewer. We have made the interval between dots long enough so that the hidden information of the image should be minimal (new Figs 2-5). Concerning the deep cerebellar nuclei (DCN), it is difficult or impossible to define its extent with the immunostaining of the present study. (Corl2 does not label DCN neurons. FoxP2 labeled a small population of DCN neurons. Pcdh10 labels a population of DCN neurons and Purkinje cell axons. EphA4 labels presumably afferent axons.) However, we referred to other immunostainings of nuclear markers at corresponding cutting levels and have added approximate boundaries of the DCN in schemes (new Figs 2-5).

3. Please check Tables carefully for accuracy and mutual consistency. For examples:

- Table 1: no reference for the anti-rat Igg

- Table 2: va1-2 is listed under ’D", but va2-4 is listed under ‘c-l’. There is no it1 in this table

- Table 3: 0-100% of what?

- Table 2 lists ic3 under ‘CM’ but, but the same ic3 is listed under “CL” in Table 4

Response: We have added the reference of the antibody, information in Table 3. We also corrected those mistakes in the tables. Concerning “it1", it was missing in Fujita et al., 2012. We adopted their nomenclature.

4. Specific words used multiple times in confusing ways:

- “then” (p14, 9 lines from bottom; p17; 14 lines from bottom; p18, 6 lines from bottom"

Because ‘then’ has temporal associations, I got confused reading the above sentences because the term “then” was used instead of “thus", or “it follows”.

Response: In these places, “then” was replaced by “on the contrary", “besides” and “consequently”.

- “nearly lacking", “near lacked”. (at least 12 useages throughout the text)

This is an awkward phrase that is difficult to parse. “nearly” makes it more confusing because near implies physical proximity. ‘Mostly lacked’ would be better, but if the authors could just establish explicility that these regions are predominantly devoid of PCs born on specific days, they could use a simpler term, eg “E10.5 PC sparse region”.

Response: We quantified the number of E10.5 PCs in the c-l cluster (1/89 at E14.5, and 12/700 at adult) (page 10, line 350 and page 16, line 536). We replaced “nearly lacked” with “mostly lacked” in some places and with “E10.5-PC-sparse” in the rest of the places.

5. pcdh10 is not defined in the Abstract, nor is its relevance or the specific focus on the ‘c-l’ cluster made clear.

Response: We have removed “Among E14.5 PC clusters, the c-l (central-lateral) cluster which lacked E10.5-born PCs divided into five c-l lineage clusters. They separately migrated underneath other clusters and positioned far apart mediolaterally as well as rostrocaudally by E17.5” (Abstract, page 2, line 52-53).

6. Abstract line 6-8 is not clear, especially “that supports the topographic connections and supports the functional localization”. What does this mean? (what does it mean to ‘provide a topographic connection’ or to ‘support a functional localization’?

Response: We revised and clarified the abstract “This somatotopy is conspicuous in the C1/C3 module, which is demarcated within the cerebellar compartmentalization as the multiple zebrin-negative and weekly-positive stripes in dual paravermal areas in anterior and posterior lobules.” (Abstract, page 2, line 41-43).

7. Significance statement is very clearly written. Is the 2nd line from the end an overstatement? This paper focused on the CL cluster, not “all of the clusters in later stages”.

Response: We clarified the differentiation of lineage of all clusters between E14.5-E17.5, and the special migration of lineage of the c-l cluster. Therefore, we think the current sentence is better. We have not changed this sentence.

8. Introduction: The statement that the C1/C3 module underlies the anterior-posterior representation of dual somatosensorymotor function seems overgeneralized. Don’t the vermis and hemispheres also have this dual somatotopy? Please clarify whether this is restricted to C1/C3.

Response: We have rewritten this and a preceding sentence to prevent overgeneralization: “Because the C1/C3 module represents the main part of the cerebellar somatotopic area as mentioned above, the anteroposterior separation of the C1/C3 module (Fig. 1B) may be the anatomical correlate for the anteroposterior dual representation of somatotopy observed in animal and human cerebellums (Snyder et al., 1950; Stoodley et al., 2012; Guell et al., 2018). “ (Introduction, page 3, line 97-101).

9. Methods: Please define “an appropriate combination” of pseudocolor - what is appropriate?

Response: We have omitted “appropriate” here: “A combination of pseudo-colors were applied to fluorescent images in figures.” (Methods, page 7, line 223-226).

10. Results line 3: “developmental transformation has been hardly clarified”. ‘hardly’ is not easy to understand. Presumably this means ‘few studies have focused on the developmental transformation’. But, what about all of the papers previously published by the authors? Please clarfiy what has and has not been clarified in prior studies so we can understand the specific questions addressed in this one.

Response: We have rewritten this sentence by referring our previous study clearly: “..., the development of embryonic PC clusters has not been fully clarified before E17.5 except for the Pcdh10-positive areas which have been tracked in our previous study (Vibulyaseck et al., 2017)” (Results, page 8, line 264-266).

11. Results page 9, end of first section. The discrepancy with previous work from this lab raises a concern about the conclusions from this and previous studies - which ones are accurate? How many clusters are there 50, 37, or some arbitrary number based on molecular expression?

Response: As explain in the general comment section, the reason we changed the number from 54 to 37 is technical rather than scientific. Essential observations in the present study were the same as in the previous study. We have fully explained why we changed the number of E17.5 PC clusters from 54 to 37 in the revised manuscript: “Although E17.5 cerebellum contains 54 clusters identified with detailed analyses (Fujita et al., 2012), this study focused on more qualitative distinction of clusters to facilitate analyses and simplify description. Namely, neighboring clusters that had only slightly different molecular expression profiles and/or not clearly separated from one another by intercalating PC-free gaps were combined. For example, our cluster vp1-2 includes clusters vp1 and vp2 of Fujita et al. (2012). We combined 11 sets of two or three neighboring E17.5 clusters into single clusters, resulting in a total of 37 clusters in place of 54. We adopted the nomenclature (Table 3) from Fujita et al. (2012) to designate F17.5 clusters in the present study. “(Results, page 9, line 307-315).

12. Results birthdate section - please refer to the corresponding Table here

Response: Table 2 is cited two times in this section (Results, page 11, line 364, 386).

13. Results p 11, line 4. Doesn’t the inclusion of 2 or more birthdates in each cluster make it more difficult (not easier) to track clusters using birthdating? Perhaps you mean that the exclusion of specific birthdates in some clusters made it easier to distinguish them from neighboring clusters.

Response: We have clarified the sentence “In sum, distribution patterns of birthdate-specific PCs were tightly linked with the PC clusters at E14.5. Consequently, the birthdate-specific labeling of PCs were expected to be a useful tool to track the lineage of the 14.5 PC clusters.” (Results, page 11, line 383-385).

14. Results p 11, last line: E17.5 is used redundantly here

Response: We have deleted “at E17.5” in this sentence: “At E17.5, the m lineage clusters were located in the most medial area (blue, vt1, vt2, vt3, vc1, vp1-2, in Fig. 7D4), whereas ...” (Results, page 12, line 419-421).

15. Results p 14, 3 lines from end: “...PCs, which extend the dendritic arbor....”. This is confusing

Response: We have rewritten this sentence: “Some tdTomato labeling in the molecular layer inside the circumscribed areas indicated the presence of labeled PC dendrite (left magenta labeling in the inset in Fig. 10B1). However, the number of labeled somata that were located within the section (arrowheads in Fig. 10B1-4) was rather small...”. (Results, page 15, line 532-539).

16. Results p 15, paragraph “in the central...” - what is the point of this paragraph?

Response: In the revised manuscript, we have divided the preceding paragraph into three paragraphs. The first sentence of this paragraph was rewritten “In the rest of cerebellar lobules (lobules VI-X, crus I, crus II, paramedian lobule, copula pyramidis, paraflocculus and flocculus), several areas were E10.5-PC-sparse and identified as the fate of the c-l clusters”. Therefore, it is now clear that we describe expression patterns of different lobules systematically in this and preceding paragraphs (Results, page 16, line 540-542).

17. Discussion, p17 -6-8 lines from bottom: Too many useages of “or” in this sentence - please divide into more sentences.

Response: This sentence has been divided into two sentences in the revised manuscript (Discussion, page 19, line 662-665).

17. Discussion p17 last lines - please comment on role of deep cerebellar nucleus in PC development

Response: We have added sentences to describe possible involvement of the deep cerebellar nucleus in the PC cluster development: “Since PC compartments are topographically connected with subareas of the cerebellar nuclei and inferior olive (Ruigrok et al., 2015), the development of the cerebellar modules may depend on the mutual development of the compartments in the cerebellar cortex, cerebellar nuclei, and inferior olive. Indeed, a genetically-induced defect in the developing cerebellar nuclei produces malformation of the cerebellar cortex (Willett et al., 2019). However, the development of compartmentalization of the cerebellar nuclei or inferior olive has not been clarified yet to the level comparable to the fine compartmentalization shown in the cerebellar cortex (Fujita et al., 2012; Fujita et al., 2020)” in the revised manuscript (Discussion, page 20, line 677-685).

18. Please implications of climbing fiber collaterals to anterior and posterior regions that share developmental origin.

Response: A similar comment was also raised by the other reviewer. We have added sentences to describe the implications of climbing fiber axon collaterals to anterior and posterior regions in the Discussion: “Single climbing fiber axons typically branch rostrocaudally and innervate both the anterior and posterior parts of the same module (Sugihara et al., 2001; Fujita and Sugihara, 2013). Axonal projections of PCs in the anterior and posterior parts of the same module converge on the same small area in the cerebellar nucleus (Sugihara et al., 2009). Such a rostrocaudal relationship in axonal projections is understandable by supposing the same axonal guidance cues (Sillitoe et al., 2009, 2010) expressed by the pair of rostral and caudal PC clusters that originated from the same early cluster.” (Discussion, page 19, line 670-677).

19. Discussion p18: “dual representation of somatosensorymotor function”. What do you mean by function here?

Response: We have replaced this phrase with “dual positioning of somatotopic areas in the cerebellar cortex” in the revised manuscript (Discussion, Page 19, line 695).

20. Discussion p18 “ The present results propose a hypothesis...” Please state the alternative hypotheses, and clarify whether your results are consistent with your hypothesis or not.

Response: In the middle of the revision process, we wrote the alternative hypothesis accordingly. However, we have eventually omitted the alternative hypothesis description, since our study is a descriptive study of the normal anatomy and development and it does not employ any intervention procedures. The alternative hypothesis description seemed to complicate the contents of the Introduction (and Discussion) rather than improving readability and comprehension. Even though we have not written the alternative hypothesis, we think we have shown that the results support our hypothesis well in the revised manuscript.

The end of this paragraphs contains several caveats that seem to require some expertise to understand. Why the use of “however” for the D0 module statement?

Response: In this part, we revised sentences, by changing the order of sentences and changing phrases: “The most lateral part of the c-l cluster formed a small part of zebrin-negative stripe 5-//6- or the D0 module (Sugihara et al., 2004). The D0 module is the somatosensorimotor module akin to the C1/C3 modules and containing the area involved in the eye-blinking reflex (Attwell et al., 2001). According to the present results most parts of the D0 module, including the entire caudal parts of the D0 module in the posterior cerebellum, seem to originate from different clusters.” (page 21, line 719-725) Therefore, we think sentences are easier to read and meaning is clearer than before.
